# Metal Exchange in the Interprotein Zn^II^‐Binding Site of the Rad50 Hook Domain: Structural Insights into Cd^II^‐Induced DNA‐Repair Inhibition

**DOI:** 10.1002/chem.201904942

**Published:** 2020-01-30

**Authors:** Michał Padjasek, Maciej Maciejczyk, Michał Nowakowski, Olga Kerber, Maciej Pyrka, Wiktor Koźmiński, Artur Krężel

**Affiliations:** ^1^ Department of Chemical Biology Faculty of Biotechnology University of Wrocław Joliot-Curie 14a 50-383 Wrocław Poland; ^2^ Department of Physics and Biophysics Faculty of Food Science University of Warmia and Mazury in Olsztyn Oczapowskiego 4 10-719 Olsztyn Poland; ^3^ Faculty of Chemistry Biological and Chemical Research Center University of Warsaw Żwirki i Wigury 101 02-089 Warsaw Poland

**Keywords:** cadmium, DNA damage, Rad50, zinc, zinc hook

## Abstract

Cd^II^ is a major genotoxic agent that readily displaces Zn^II^ in a multitude of zinc proteins, abrogates redox homeostasis, and deregulates cellular metalloproteome. To date, this displacement has been described mostly for cysteine(Cys)‐rich intraprotein binding sites in certain zinc finger domains and metallothioneins. To visualize how a Zn^II^‐to‐Cd^II^ swap can affect the target protein's status and thus understand the molecular basis of Cd^II^‐induced genotoxicity an intermolecular Zn^II^‐binding site from the crucial DNA repair protein Rad50 and its zinc hook domain were examined. By using a length‐varied peptide base, Zn^II^‐to‐Cd^II^ displacement in Rad50’s hook domain is demonstrated to alter it in a bimodal fashion: 1) Cd^II^ induces around a two‐orders‐of‐magnitude stabilization effect (log K12ZnII =20.8 vs. log K12CdII =22.7), which defines an extremely high affinity of a peptide towards a metal ion, and 2) the displacement disrupts the overall assembly of the domain, as shown by NMR spectroscopic and anisotropy decay data. Based on the results, a new model explaining the molecular mechanism of Cd^II^ genotoxicity that underlines Cd^II^’s impact on Rad50’s dimer stability and quaternary structure that could potentially result in abrogation of the major DNA damage response pathway is proposed.

## Graphical abstract

**A genotoxic swap**: Zn^II^‐to‐Cd^II^ displacement in the intermolecular Zn^II^‐binding site of the DNA‐binding protein Rad50 drastically elevates the stability of the metal–protein complex and disrupts its overall assembly.

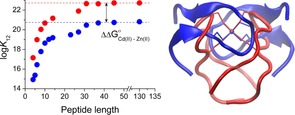

## Introduction

Cadmium is a well‐defined nephrotoxic, pneumotoxic, osteotoxic, and cardiotoxic agent and, on top of that, a strong carcinogen, a feature probably stemming from its extensive indirect genotoxicity.^1^, ^2^, ^3^, ^4^, ^5^ Its detrimental effects on human health are known, but a clear mechanism connecting cadmium's intake and its indirect genotoxicity is still elusive, which seems to be dictated by a multivalent effect occurring as a result of binding of the Cd^II^ ion by its cellular targets.^6^ Cd^II^ as a softer Lewis acid than Zn^II^ readily displaces it in cysteine(Cys)‐rich zinc‐binding proteins and biomolecules, which solitarily introduces an enormous toxic effect inside a cell.^7^ Zn^II^ fluctuations, which impact the exchangeable zinc quota,^8^ interfere with redox homeostasis, and deregulate cellular metalloproteome.^9^ Furthermore, because Cd^II^ binds preferentially to thiol‐containing and other nucleophilic ligands, it interacts with a wide spectrum of redox signaling and reactive oxygen scavenger proteins, intensifying Zn^II^‐displacement effects.^10^ Besides generating a pool of displaced metal ions, Cd^II^ interferes with its target's biophysical properties, namely stability, flexibility, and overall structure.^11^, ^12^ Structure‐interfering properties of Cd^II^ binding to Zn^II^‐containing proteins remain controversial because for decades Cd^II^ was used as a Zn^II^‐isostructural spectroscopic probe for Zn^II^‐binding proteins in UV spectrophotometric studies and ^113^Cd NMR spectroscopy.^13^, ^14^, ^15^, ^16^, ^17^ Although Cd^II^ and Zn^II^ ionic radii are relatively similar and displacement is usually one‐to‐one, in terms of stoichiometry and coordination sphere, their binding to a target protein could potentially generate a different structural outcome. The presented scientific problem of Zn^II^‐to‐Cd^II^ substitution and its impact on zinc‐binding proteins have been extensively studied; however, all published data are focused on intramolecular sites, mostly of zinc finger domains and metallothioneins. Effects of this substitution are not uniform and involve no, or very slight, changes in structure and function, like in the case of Sp1,^18^ Tramtrack,^19^ SUP37,^20^ or Ros87,^21^ as well as significant alterations of the domain fold, in the case of XPA,^5^, ^22^, ^23^ p53,^24^ or MTF‐1.^25^

**Figure 1:**
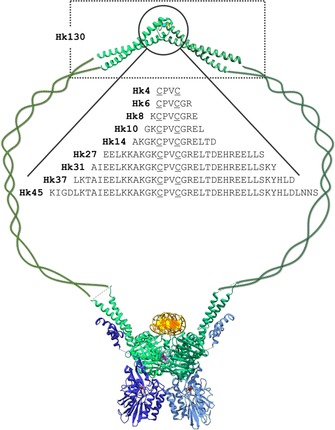
Architecture of the MR(N/X) complex with Rad50 fragments (Hk4–Hk130) investigated in this study. Green, blue, and yellow represent two Rad50, two MR, and one DNA molecule, respectively. Structural representation is based on the crystal structure of a central 112‐amino‐acid‐long Rad50 (*P. furiosus*) fragment with Hg^II^ (PDB code: 1L8D) and the globular apex of a MR complex from *Methanocaldococcus jannaschii* bound to DNA (PDB code: 5DNY).

Herein, we aim to illustrate for the first time how this phenomenon affects the intermolecular zinc‐binding site by investigating Cd^II^ binding to the central fragment of *Pyrococcus furiosus* (*P. furiosus*) Rad50 protein. Rad50 is a constituent of the MR(N/X) (Nbs1/Xrs2 is distinctive for eukaryotes) complex responsible for double‐stranded DNA damage signaling and repair. Active as a dimer, it consists of two protomers with a typical structure for the SMC protein family: a very long antiparallel coiled‐coil (cc) segment that connects two apexes of the protein (i.e., the globular DNA‐binding ATPase domain), and the zinc hook domain responsible for dimerization by formation of a tetrathiolate Zn(CXXC)_2_ coordination sphere (Figure 1).^26^, ^27^ The overall dimeric assembly of the MR(N/X) complex is not well‐defined, and data from electron and atomic force microscopy present multiple conformations of the complex ranging from open‐circular‐like to closed‐rod‐shape‐like.^26^, ^28^, ^29^, ^30^ Such alternating behavior seems to be a common feature of the SMC protein family and, for Rad50 in particular, could potentially allow mechanically driven functional diversification; for example, switching between pathways of DNA damage repair. This process, however, could only be accommodated through specific flexibility of both globular and zinc hook apexes, the former of which has already been documented^31^ but the latter remains hypothetical. We hereby present an insight into molecular bases of Cd^II^ toxicity and indirect genotoxicity, taking into account the impact of Cd^II^ on Rad50’s dimer stability and quaternary structure that could potentially result in abrogation of the major DNA damage response (DDR) pathway (and subsequent DNA lesions, chromosomal aberrations, and carcinogenesis).

Previous studies on the central fragment of the zinc hook domain of the Rad50 protein from *P. furiosus* demonstrated that this fold forms an extremely stable complex with Zn^II^.^32^, ^33^ The molecular basis of this high stability has been analyzed by using a set of hook model peptides (Hk) containing a ‐CPVC‐ binding motif with increased peptide flanks from both ends (Hk4–Hk45).^32^ The results indicated that the formation of a hook homodimer (Zn(Hk)_2_) occurs in a stepwise manner starting from the formation of the ZnHk complex, followed by association of another Hk molecule. The increased difference in stability of the two complexes with rising peptide length favors the formation of the Zn(Hk)_2_ species. The existence of an equimolar complex being in equilibrium with a dimer could be of high importance and suggests a possibility of readily exchangeable Rad50 molecules during cellular processes, which is believed to be the requirement for MRN native functions.^34^, ^35^ Keeping in mind the toxic effect of Cd^II^ on zinc finger domains of transcription factors and other zinc–sulfur proteins associated with DNA processing, we aimed to examine the impact of Cd^II^ binding on the Rad50 protein using a well‐described model from *P. furiosus*,^32^, ^33^ which would be the first intermolecular zinc‐binding site analyzed in terms of Cd^II^ attack. The aim of this study was threefold: 1) to demonstrate how strongly Cd^II^ ions bind to the Rad50 protein, 2) to show whether or not Cd^II^ ions are able to replace Zn^II^ in the cellular timescale, and finally, 3) to analyze how Cd^II^ binding affects the Rad50 hook structure in a way that could translate into functional alterations of Rad50 and the entire MRN complex.

## Results and Discussion

In the first stage of the study, we examined whether Cd^II^ is capable of binding in the same stoichiometric model as Zn^II^ and whether it forms more stable complexes allowing Cd^II^ to exchange Zn^II^ from the hook domain. To assess if the structure and stability of Cd^II^ complexes rely on a similar structural basis to that of Zn^II^, we used a series of zinc hook (Hk) peptides ranging in length from 4 to 130 amino acid residues (Hk4–Hk130) (Figure 1). Peptide Hk45 has been recognized as a fragment which, as a Zn^II^ complex, possesses all residues that form intermolecular contacts and contains both α‐helical and β‐hairpin regions of the domain.^33^ Peptides Hk4, Hk6, Hk10, and Hk14 were used to investigate metal‐coupled formation of the β‐hairpin. The remaining peptides (Hk27, Hk31, and Hk37) were applied for investigation of any important changes occurring in the vicinity of the region between the β‐hairpin and the elongated domain (about 45 amino acid residues). The longest Rad50 protein fragment, containing 130 amino acid residues, was used as a Rad50 protein model containing a long coiled‐coil fragment to examine whether effects occurring in minimal fragments are transferred to other protein regions and vice versa.

### Spectroscopic analysis of Cd^II^ binding to the hook motif

**Figure 2:**
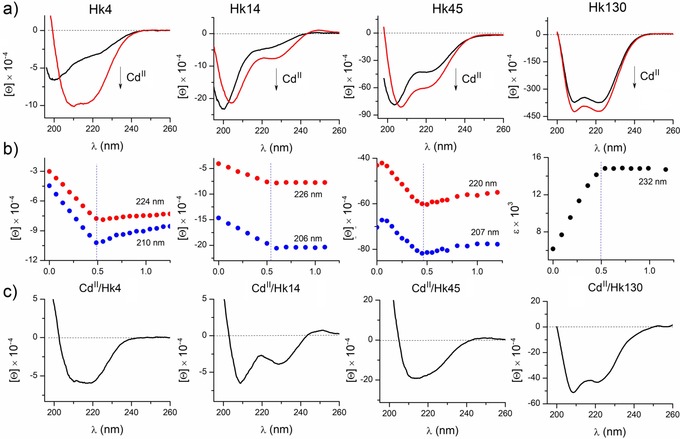
Cd^II^ binding to selected hook peptides recorded by CD and UV/Vis spectroscopy in 20 mm Tris‐HCl buffer, pH 7.4, *I*=0.1 m. a) CD spectra of the apo form (black line) and the complex at a Cd^II^‐to‐Hk peptide molar ratio of 0.5 (red line). b) Dependence of the ellipticity (Hk4, Hk14, Hk45) and absorbance (Hk130) at listed wavelengths on the Cd^II^/Hk molar ratio. c) Differential CD spectra of hook peptides obtained by the subtraction of the spectra of free peptides from the spectra of Cd(Hk)_2_ complexes. [Θ] and *ϵ* refer to molar ellipticity (in deg cm^2^ dmol^−1^) and molar absorption coefficient (in m ^−1^ cm^−1^), respectively.

Binding of Cd^II^ to hook peptides was monitored using several spectroscopic methods including spectrophotometry and spectropolarimetry for acetylated and amidated peptides, as well as fluorimetry and fluorescence anisotropy in the case of fluorescently labeled peptides. Far‐UV circular dichroism (CD) titrations, performed by the addition of Cd^II^ to metal‐free peptides at pH 7.4 in the presence of tris(2‐carboxyethyl)phosphine hydrochloride (TCEP) (used as a non‐metal‐binding reducing agent),^36^ demonstrated extensive conformational changes for most investigated peptides upon Cd^II^ coordination (Figure 2 a and Figure S1, Supporting Information). Spectrophotometric titration in the same region showed formation of a band with the maximum between 230 and 250 nm. This band corresponds to ligand‐to‐metal charge transfer (LMCT) events, and it is typical for CdS_4_ metal centers found in tetrathiol‐containing ligands, such as Zn(Cys)_4_ (Figure S2, Supporting Information).^37^, ^38^, ^39^ Both CD and UV/Vis spectra demonstrate a sharp inflection in the titration curves at a 1:2 Cd^II^‐to‐peptide molar ratio, which confirms the formation of predominantly Cd^II^‐mediated homodimers: ML_2_ complexes (Figure 2 b). However, in the case of Hk27, Hk31, and Hk45, the formation of CdHk (ML) complexes also occurs. The largest difference in stability between ML and ML_2_ complexes is observed for Hk27, which is reflected by two visible inflection points, whereas the Hk6, Hk10, and Hk14 peptides demonstrate the strongest tendency to form only dimeric species, similarly to the study of Zn^II^ complexes^33^ (Figure 2 and Figure S2, Supporting Information). In the case of Hk130, the CD spectra changes slightly (Figure 2 a), which complicates stoichiometric analysis. However, spectrophotometric titration in the UV range clearly shows the formation of the Cd(Hk130)_2_ homodimer, which is the predominant species (Figure 2 b and Figure S3, Supporting Information). Depending on the examined peptide, the Cd(Hk)_2_ complex adopts different conformations, as indicated by the distinct CD spectra. The structural changes obtained during Cd^II^ coordination can be compared qualitatively based on the differential spectra obtained through the spectral subtraction of free Hk peptide from their complexes with the metal ion (Figure 2 c). Both types of spectra, together with their molar ellipticity, indicate the formation of a β‐hairpin‐like structure in the case of Cd(Hk14)_2_ and mixed β‐hairpin and helical structures in the case of Cd(Hk45)_2_. The case of the longest Hk130 peptide is certainly different due to a long helical structure (more likely being a part of coiled‐coil structure) that is already present in the metal‐free form. The Cd^II^ binding to this protein fragment causes only small changes in the CD spectra, indicating additional structurization in the central metal binding part and parallel stabilization of the coiled‐coil structure. Although the peptide backbone contribution is evident, one has to account for the Cd–S LMCT contribution in the overlapping region. Nonetheless, the backbone‐related transitions presented herein are around 10–20 times stronger; hence, the Cd^II^‐binding contributions are significantly overshadowed and do not influence the qualitative assessment.^37^

**Figure 3:**
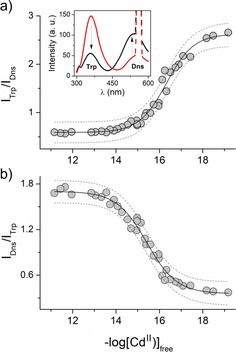
Cd^II^ binding to 5 μm fluorescently modified Hk14 investigated fluorometrically under controlled free Cd^II^ conditions (HEDTA, EDTA, and TPEN competition) in 50 mm 4‐(2‐hydroxyethyl)piperazine‐1‐ethanesulfonic acid sodium salt (HEPES) buffer, pH 7.4, *I*=0.1 m. a) Tryptophan‐to‐dansyl intensities ratio (*I*_Trp_/*I*_Dns_) changes. The inset demonstrates the emission spectra of free (black line) and Cd^II^‐bound (red line) Hk14. b) Dansyl‐to‐tryptophan intensities ratio (*I*_Dns_/*I*_Trp_) changes. Ratios were fitted to Equations (2) and (3), according to a protocol by Pomorski et al.^52^ Dashed lines represent a plot confidence of 95 %.

Metal‐coupled β‐hairpin formation in the middle of the zinc hook upon Cd^II^ complexation has been demonstrated by using a minimal N‐terminally dansylated Hk14 peptide with a tryptophan residue placed at its C‐terminus. Figure 3 (inset) shows the peptide's emission spectra when the tryptophan (Trp) residue is excited at 290 nm. Emission spectra demonstrate a fluorescence resonance energy transfer (FRET) effect between Trp and dansyl (Dns) residues, but the efficiency shows that both donor and acceptor are far away in the metal‐free peptide, indicating the disorganized nature of the Hk14 peptide.^32^ When Cd^II^ is bound, and the homodimer is formed, the intensity of Trp significantly decreases while the intensity of the acceptor increases, demonstrating a substantial efficiency increase.^40^, ^41^ Taking into account the Förster radius of the Trp–Dns FRET pair, being between 21 and 24 Å, it is likely that this FRET efficiency change may be attributed to the proximity of both fluorophores around this range or rather below it.^42^ Indeed, the molecular dynamics simulation of Hk14 in the Cd^II^ complex (see Supporting Information for more details) shows that the estimated average distance between donor and acceptor in the Cd^II^ complex is (12.7±2.5) Å. Similar FRET efficiency change upon metal binding has been observed for the Hk14 peptide and several of its mutants upon Zn^II^ complexation.^32^ However, the change occurring in the case of Cd^II^ is much more pronounced than that of its Zn^II^ counterpart, suggesting either more compact structure of a protomer or smaller distance between the N‐ and C‐termini of a single protomer or between opposite termini of both protomers in Cd(Hk)_2_. The hook structure, even in its minimal fold, is highly sensitive to any close‐occurring changes. Our previous study showed that alanine scanning in the Hk14 sequence may affect the FRET effect significantly, indicating that even one non‐metal‐binding residue substitution may impact the hook's global structure.^32^ Moreover, changes occurring in the further region of the full domain also affect the structure and stability of formed complexes; this has been shown by using a longer peptide model as well as in vivo tests on the full MRX complex.^43^ Mutations of the hook domain's Cys‐neighboring amino acids alter DNA damage repair and signaling functions of the entire complex, proving that alteration of the hook motif is transferred to the globular parts of the MRN complex, presumably through the coiled‐coil region, and render it abrogated.^44^, ^45^

To thoroughly investigate the Cd^II^ ion's impact on the Rad50’s zinc hook domain's assembly, we turned to anisotropy decay analysis. Measuring time‐resolved anisotropy change provides vast analytical opportunities and gives insights into protein dynamics, dimensions, as well as protomer arrangement in oligomeric species. N‐terminally FAM‐labeled (FAM=5(6)‐carboxyfluorescein) *P. furiosus* Rad50 Hk14 and Hk45 were subjected to an anisotropy decay experiment either as apo forms, Zn^II^/Cd^II^‐loaded, or analyzed after overnight incubation in a set of metal buffers. We focused our attention on one major parameter derived from anisotropy decay analysis: rotational correlation time [τ_r_, Eq. (1)], which delivers information about molecular dimensions in terms of their diffusion capability in a solvent, for which *I* is intensity, *r*_inf_ is residual anisotropy, and τ${}_{r{}_{1}}$ and τ${}_{r{}_{2}}$ and *B*_1_ and *B*_2_ are rotational correlation time and amplitude, respectively, for both exponentials. Because the τ_r_ parameter describes how fast the fluorophores rotate, thus decreasing their anisotropy, it is directly correlated with the hydrodynamic radius of the emitting molecules.^42^

(1) $$I_{r}\left(i\right)=r_{\inf}+B_{1}\exp\left(\frac{-i}{\tau_{r_{1}}}\right)+B_{2}\exp\left(\frac{-i}{\tau_{r_{2}}}\right)$$

**Figure 4:**
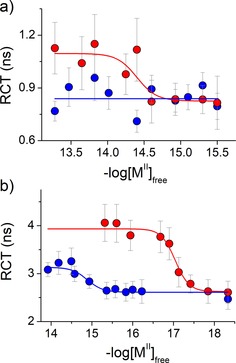
Rotational correlation time (RCT) changes of 500 nm FAM‐labeled a) Hk14 and b) Hk45 presented as a function of the molar concentration of free Zn^II^ or Cd^II^ (−log [M^II^]_free_) in HEPES buffer pH 7.4. Gray and red circles represent data from Zn(Hk)_2_ and Cd(Hk)_2_ experiments, respectively. Error bars represent standard deviation errors from two‐exponential fitting of reconvolution anisotropy spectra (see the Supporting Information for more details).

The rotational correlation time parameter differs significantly for Zn^II^ and Cd^II^ complexes, which is clearly visible from plots of τ_r_ against a range of free metal ion concentrations (Figure 4). These results demonstrate that τ_r_ for both peptides increases with free metal ion concentration, as a result of metal‐ion‐induced dimerization, but the increase from metal‐free to metal‐bound form is around 30 % higher for a Cd^II^‐binding event than that of Zn^II^. The experiment suggests that Hk14 and Hk45 molecules in Zn^II^ and Cd^II^ complexes present different dimer arrangements with different hydrodynamic diameters, the former being smaller than the latter.^42^, ^46^ Taking into account that the Cd^II^ ionic radius (109 pm) is slightly larger than the Zn^II^ radius (88 pm),^10^ this trait feels natural. However, it seems that swelling of the Rad50 dimer interface caused by incorporating a bigger metal ion is transferred further down through both protomers, changing the overall quaternary structure of the analyzed dimers.

Our approach possesses one major intrinsic obstacle that prevents precise volumetric measurement of analyzed dimers; as a result of the homo‐labeling setup, homo‐FRET events are very likely to occur between two FAM‐labeled N‐termini of Rad50 dimers, which results in an additional route of anisotropy decay. We speculate that homo‐FRET transfer is represented as an additional decay time; however, it is too fast to allow quantitative assessment of differences between alternatively composed hook dimers. To resolve whether a homo‐transfer's impact on anisotropy decay overshadows differences in molecular volume, we analyzed initial anisotropy values for both complexes. Initial anisotropy (not fundamental anisotropy, which is constant for 5(6)‐carboxyfluorescein and equals 0.38) would be significantly affected by the homo‐FRET phenomenon, making it an ideal control parameter to ascertain the validity of this approach.^42^ Interestingly, initial anisotropy values for complexes with Zn^II^ were a little higher than those of complexes with Cd^II^, suggesting homo‐FRET of higher efficiency for the latter (Figure S4, Supporting Information). This means that differences in rotational correlation time are probably more pronounced, and the τ_r_ value for Cd(Hk)_2_ may be in fact higher than what we have observed.

### Determination of stability constants of Cd^II^ zinc hook complexes

**Figure 5:**
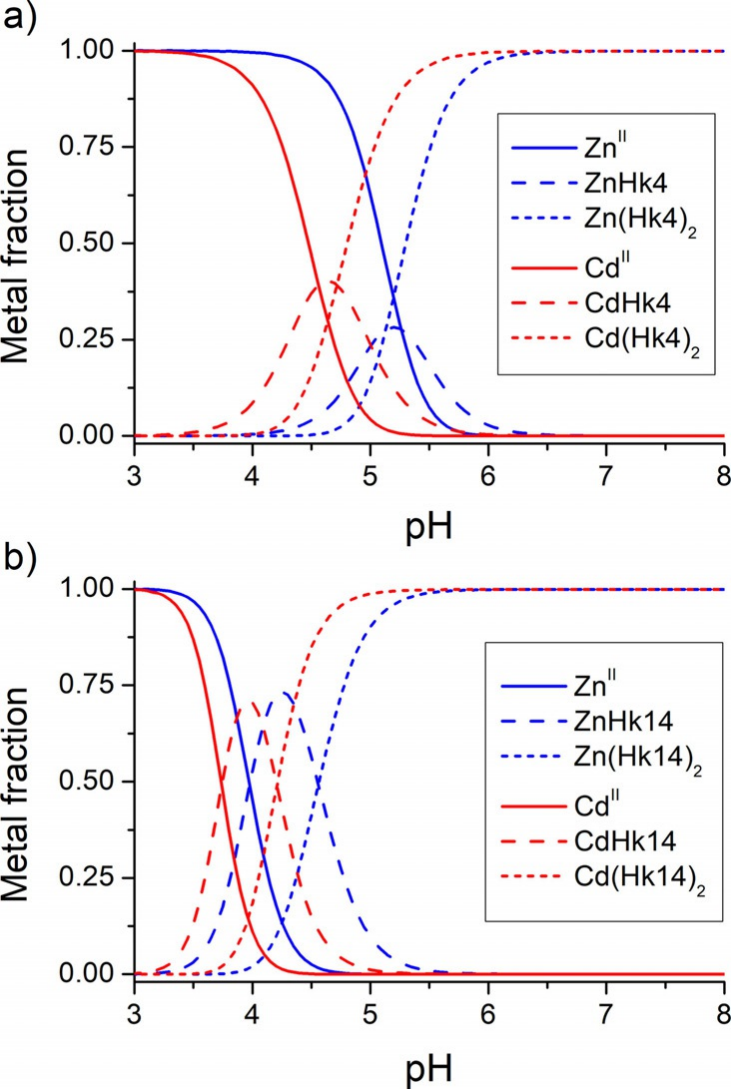
Molar species distribution of Cd^II^ and Zn^II^ complexes with a) Hk4 and b) Hk14 peptides calculated based on protonation and stability constants determined potentiometrically. Hk peptides and metal ion concentrations were set as 400 and 200 μm, respectively, as in the potentiometry experiments.^32^

As demonstrated in the spectroscopic studies, Cd^II^ titration curves of Hk peptides demonstrate isotherms of complex formation with a sharp inflection point, which clearly indicate that affinity of the studied hook peptides towards Cd^II^ is high, and direct spectroscopic stability constant determination would not be feasible without risk of their underestimation.^47^, ^48^ Therefore, to determine actual stability constant values of the formed Cd^II^ complexes, several approaches were used. The first one and the most informative regarding the stoichiometric model and cumulative stability constants (formation constant *β_ijk_* of the M_*i*_H_*j*_L_*k*_ complex, which includes the protonation state of the ligands coordinated to the metal ion) was potentiometry. However, as a result of the limited number of residues with acid–base properties, which is a requirement for this method, it was applied here only for Hk4–Hk14 peptides.^49^ The obtained results confirmed that ML and ML_2_ stoichiometries of the complexes are formed during complexation and are variously protonated depending on the peptide chain length (Figure 5). Table 1 presents cumulative constants of all peptides investigated potentiometrically as well as the p*K*_a_ values of deprotonating groups. Species distribution presented in Figure 5 shows that the ML_2_ (M=Cd^II^) complex is formed at low pH (from about 4 and 3.5 for Hk4 and Hk14, respectively) and is stable up to basic pH. For comparison, Zn^II^ complexes of the same peptides are formed at around 0.5 unit higher in pH, indicating a significant difference between the two metal ions. This pH difference in dimeric complex formation is demonstrated by approximately two‐orders‐of‐magnitude lower stability constants for Zn^II^ complexes when compared to Cd^II^ ones, although this difference varies depending on the peptides’ length (Table 1).^33^

**Table 1:** Cumulative protonation and Cd^II^ stability constants (log *β_ijk_*)^[a]^ of hook peptide complexes determined potentiometrically at 25 °C, *I*=0.1 m (from KNO_3_).^[b, c]^

Species	Hook peptide (log *β_ijk_*)				
	Hk4	Hk6	Hk8	Hk10	Hk14
HL	9.372±0.03	9.01±0.04	10.65±0.02	10.64±0.04	10.80±0.03
H_2_L	17.52±0.02	16.86±0.02	19.75±0.02	19.48±0.03	20.48±0.01
H_3_L	–	–	27.57±0.03	26.85±0.02	39.19±0.02
H_4_L	–	–	31.81±0.02	31.02±0.04	36.48±0.02
H_5_L	–	–	–	–	40.89±0.03
H_6_L	–	–	–	–	44.88±0.04
CdH_2_L	–	–	–	–	33.33±0.04
CdHL	–	–	23.19±0.04	22.96±0.04	–
CdL	12.58±0.04	12.33±0.03	–	–	–
CdH_4_L_2_	–	–	–	–	65.26±0.07
CdH_3_L_2_	–	–	–	–	56.10±0.09
CdH_2_L_2_	–	–	45.86±0.02	45.20±0.05	46.80±0.04
CdHL_2_	–	–	36.61±0.03	37.66±0.06	36.55±0.03
CdL_2_	22.74±0.03	23.38±0.02	n.d.	n.d.	n.d.

[a] *β*(M_*i*_H_*j*_L_*k*_)=[M_*i*_H_*j*_L_*k*_]/([M]^*i*^[H]^*j*^[L]^*k*^), in which [L] is the concentration of the fully deprotonated zinc hook peptide. [b] Standard deviations are given as provided by SUPERQUAD calculations. [c] n.d. denotes ‘not determined’ under used conditions.

**Figure 6:**
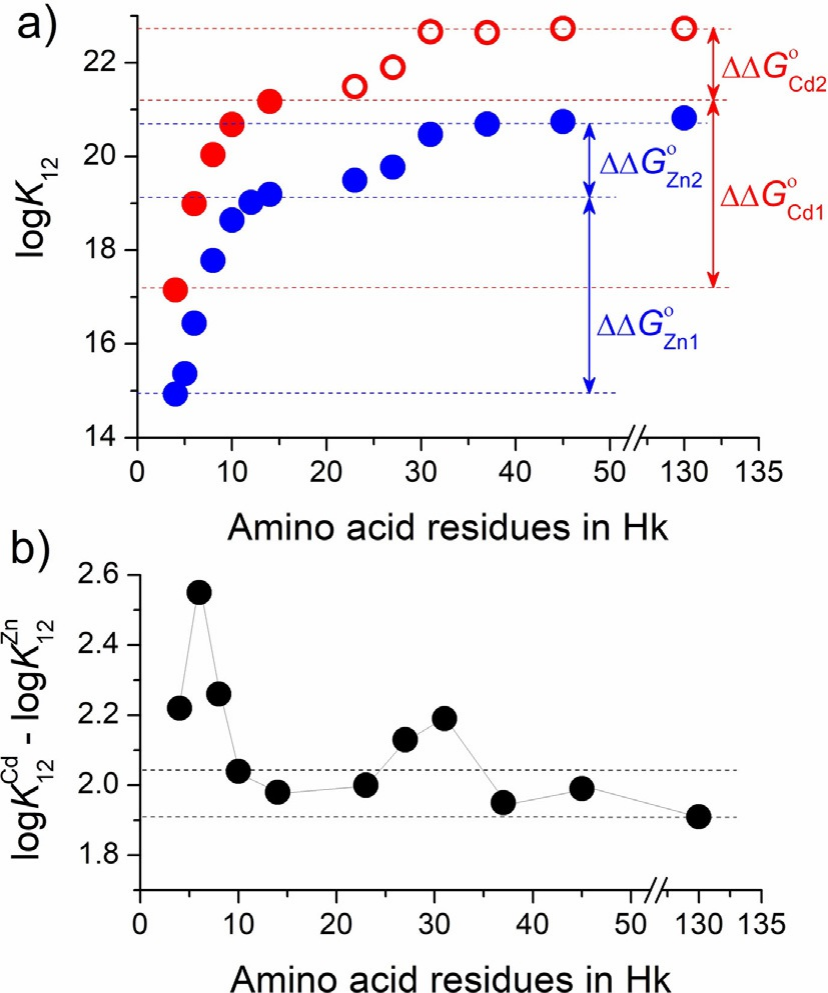
Comparison of conditional *K*_12_ constants (log *K*_12_) of Cd(Hk)_2_ and Zn(Hk)_2_ complexes at pH 7.4 varying in length of the zinc hook peptide. a) Blue circles correspond to Zn^II^ complexes determined previously.^33^ Red circles represent constants of Cd^II^ complexes determined herein potentiometrically (solid circles) and from competition with metal complexones (open circles). b) Differences in log *K*_12_ between Cd^II^ and Zn^II^ complexes. Dashed lines indicate the range of complex stability resulting only from metal‐ion exchange.

Given that cumulative stability constants are pH‐independent, the direct comparison of affinities between particular ligands or metal ions at certain pH is impossible without consideration of *K*_a_ values of metal‐free ligands, which differ between each other. To present potentiometric data in a comparable way (with conditional constants determined spectroscopically), we calculated the formation constant *K*_12_ of the Cd(Hk)_2_ complexes valid for pH 7.4 as shown below [Eq. (2)]:

(2) $$K_{12}=\frac{\left[{Cd\left(Hk\right)}_{2}\right]}{\left[{\mathrm{Cd}}^{\mathrm{II}}\right]{\left[Hk\right]}^{2}}$$

in which [Hk] is the sum of all metal‐free Hk species with variously protonated states as well as fully deprotonated species at pH 7.4 being in equilibrium in the Cd(Hk)_2_ complex. Such conditional constants demonstrate that affinity of Hk peptides towards Cd^II^ increases together with the peptide length from about 10^17^ m ^−2^ for Hk4 to about 10^21^ m ^−2^ for Hk14 (Table 2). However, the largest increase in stability is observed between Hk6 and Hk10, similarly to Zn^II^ complexes (Figure 6), suggesting that Cd^II^‐mediated folding of the β‐hairpin is the energy force that elevates stability of the hook domain, analogously to Zn^II^ complexes.^32^, ^33^

**Table 2:** Conditional formation constants (*K*_12_) of Cd(Hk)_2_ complexes and corresponding competitivity indexes (CI) obtained from potentiometric, CD, and fluorometric (FL) data. Standard error is provided only for data obtained in competition and exchange experiments. Potentiometry‐derived *K*_12_ values were calculated from data presented in Table 1.

Hook peptide	Method of determination	log *K*_12_	CI^[a]^
Hk4	potentiometry	17.15	13.85
Hk6	potentiometry	18.99	15.69
Hk8	potentiometry	20.04	16.74
Hk10	potentiometry	20.68	17.38
Hk14	potentiometry	21.16	17.86
	FL competition	21.17±0.05	17.87
Hk27	CD competition	21.96±0.07	18.66
Hk31	CD competition	22.60±0.09	19.30
Hk37	CD competition	22.64±0.03	19.34
Hk45	CD competition	22.64±0.05	19.34
Hk130	FL competition	22.73±0.05	19.43

[a] CI is the logarithm of the apparent dissociation constant of the CdL complex (Cd^II^ complex of theoretical molecule Z), such as [CdZ]=Σ_*ijk*_[Cd_*i*_H_*j*_L_*k*_] at given overall component concentrations. The concentrations of Z were set at 1 mm and those of Cd^II^ at 0.25 mm.

**Figure 7:**
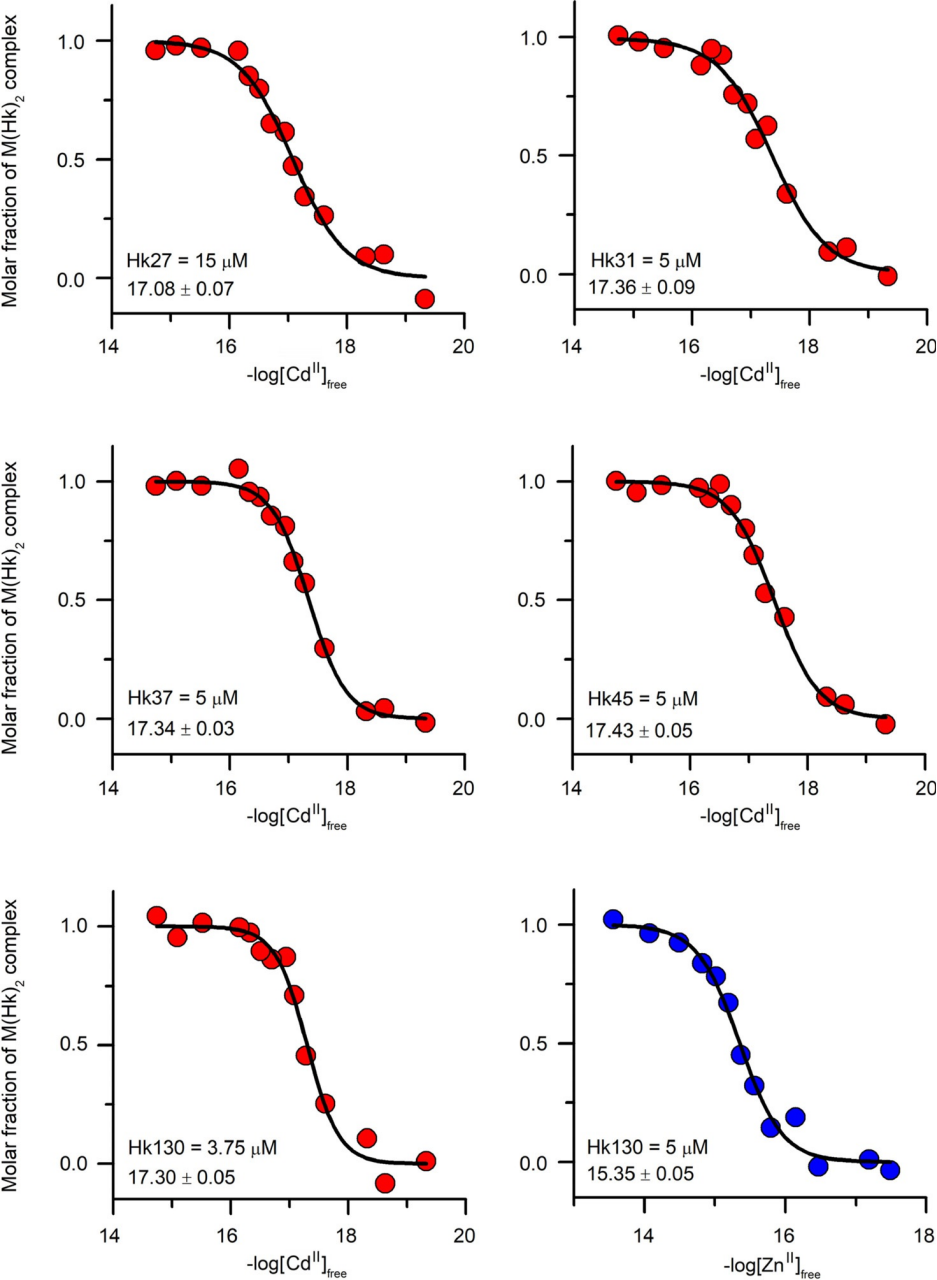
Isotherms of Cd^II^ (red circles) and Zn^II^ (blue circles) binding to Hk27, Hk31, Hk37, Hk45, and Hk130 in the presence of 25 μm metal chelators determined spectropolarimetrically or fluorometrically in 20 mm Tris‐HCl buffer, pH 7.4, *I*=0.1 m. Peptides were used at various and listed concentrations. Data were fitted to the Hill equation, and the presented values correspond to half‐saturation points (−log [Cd^II^]_free_^0.5^ and −log [Zn^II^]_free_^0.5^) used for *K*_12_ calculation (Table 2).

Although potentiometry is a good method for determination of stability constants of highly stable complexes, its application is limited to the number of acid–base groups, and it is difficult or impossible to use for proteins and their larger fragments. Therefore, to determine *K*_12_ of Cd^II^ complexes with Hk27, Hk31, Hk37, Hk45, and Hk130 peptides, we applied CD‐monitored competition with common metal complexones with well‐established stability constants, such as HEDTA, EDTA, and TPEN.^48^, ^50^ Because ellipticity changes of the Hk130 peptide are the smallest, CD data were supported by natural tyrosine fluorescence change, the intensity of which decreases upon Cd^II^ binding (Figure S5, Supporting Information). Figure 7 shows normalized ellipticity or fluorescence intensity as a function of free Cd^II^ ions, calculated using Hyperquad software based on protonation and stability constants of Cd^II^–complexone complexes.^51^ To determine *K*_12_ values, experimental data were first fitted to Hill's equation^32^ to obtain half‐saturation points (−log [Cd^II^]_free_^0.5^). In the next step, *K*_12_ values were calculated according to Equation (2), assuming that the half‐saturation point corresponds to the half of maximal concentration of Cd(Hk)_2_.^32^, ^33^ Obtained *K*_12_ values with standard deviation error are presented in Table 2 and also illustrated in Figure 6 (open red circles), in which they overlap very well with *K*_12_ values obtained from potentiometry for shorter peptides (solid red circles). These results indicate acceptable data coverage between the two methods (potentiometry and spectroscopy).

Because Hk14 was shown to be the minimal fold of extreme stability,^32^ its complex with Cd^II^ was herein investigated primarily and resulted in an additional set of competition data gathered for the fluorescently labeled Hk14 with Dns and Trp moieties. This type of modification enables assessment of wide dynamic range changes that guarantee the highest‐quality data determination. Moreover, the presence of two bands corresponding to the donor and acceptor of the FRET pair makes this peptide a sensitive ratiometric sensor of free Cd^II^ concentration and conformational changes. Figures 3 a, b demonstrate Trp/Dns (*R*_Trp_) and Dns/Trp (*R*_Dns_) ratios of intensity changes, respectively, as a function of free Cd^II^. To determine the actual −log [Cd^II^]_free_^0.5^ value from the ratiometric study, we applied our normalizing equations that are based on fixed maximal and minimal intensities of Trp and Dns bands, independently, as described elsewhere.^52^ Application of these formulae to fluorescence intensity ratios resulted in actual −log [Cd^II^]_free_^0.5^ values of 15.8 and 15.7 for *R*_Trp_ and *R*_Dns_, respectively, allowing further log *K*_12_ calculation to give 21.17. Formation constants obtained with this method are convergent with the values calculated from potentiometric data (Table 2).

### Stability comparison of Cd^II^‐substituted hook domain with other Cd^II^ complexes

Our previous results have shown that the zinc hook domain is among the most stable Zn^II^ complexes found in proteins and their natural domains. For example, the Zn(Hk45)_2_ complex is more stable than the most stable natural zinc fingers described so far, such as Sp1‐3, MTF‐1, or Zif268‐2, when competing in the micromolar range with Hk45.^33^, ^47^, ^53^ When concentrations of both protein fragments decrease, zinc fingers demonstrate a higher tendency to bind Zn^II^. Such behavior comes from the fact that the hook domain forms an ML_2_‐type complex, whereas zinc fingers and most other proteins form an ML‐type complex. Our recent results obtained on various zinc domains with different Zn^II^‐to‐protein stoichiometry, yet similar affinity, showed concentration dependence for metal‐mediated complexes.^54^ Such concentration‐dependent metal association might be an important feature for transient saturation and transient protein function. In light of these results, there are important questions of how Cd^II^ ions may affect zinc hook structure and function, and how Cd^II^ binding may affect possible transient saturation of the hook domain. To get closer to answering these questions and to properly order the cadmium hook in the stability hierarchy of Cd^II^ complexes with proteins, it is worth analyzing the literature in terms of stability data of Cd^II^ complexes of other proteins or peptide models. Table 2 presents the apparent formation constants of Cd(Hk)_2_ at pH 7.4, calculated either from potentiometric data or determined by competition with complexones. Because of the fact that the hook motif forms an ML_2_‐type complex, direct comparison of its formation constant (*K*_12_) with constants of complexes with ML stoichiometry of other ligands is impossible. To avoid this problem, we converted all formation constants present in Table 2 to the competitivity index (CI), which has been shown previously to be useful for the comparison of affinities of metal complexes with various stoichiometries.^55^, ^56^ In principle, it simplifies any stoichiometry to M_*x*_L_*y*_ under certain reactant concentrations and is valid only when comparable ligands and metal ion are present in the same concentrations.^56^ Thus, a quick comparison of CI values of Cd^II^ hook complexes and other Cd^II^ complexes found in the literature (Table 3) shows that the investigated complexes, Hk14–Hk130, are the most stable found to date. The CI values of these Cd^II^ hook complexes are two‐to‐four orders of magnitude more stable (CI=17–19) than the strongest ones with XPA zf (CI=12.8),^23^ CadC (CI=12.6),^57^ CmtR (CI=12.2),^58^ or MT2 (CI=14.4 for β‐domain and 15.8 for α‐domain).^59^ Moreover, the complexes investigated here are much more stable than the Cd^II^ complex with EDTA (CI=13.6),^60^ consensus CP1 zf (CCCC) zinc finger (CI=13.4),^61^ or short poly‐Cys peptides known to form highly stable complexes with Cd^II^, for instance Ac‐YCSSCY or Ac‐CC‐NH_2_, for which CI is 14.8 and 12.6, respectively.^62^, ^63^ This brief overview clearly demonstrates that the zinc hook domain with substituted Cd^II^ must exhibit unique stabilization effects that elevate thermodynamic stability. Molecular reasons for such stability are discussed below.

**Table 3:** Affinities of selected low‐molecular‐weight ligands, peptides, and proteins for Cd^II^ collected across the literature that form highly stable complexes. Stability constants were determined under various conditions, which are listed. If not specified, values refer to pH 7.4. RT, pHT, CC, and n.c. refer to reverse titration, pH titration, competition with metal chelator, and not calculated, respectively. CI values derived from formation constants presented as ‘not calculated’ (n.c.) were determined from published log *β_ijk_* data from potentiometric analyses.^38^, ^39^, ^62^, ^63^, ^83^

Ligand^[a]^	Reference	Method of determination	Conditions	Stoichiometry of the complex	Formation constant (log *K*)	CI
EDTA	60	potentiometry	100 mm KNO_3_	ML	13.6	13.6
HEDTA	60	potentiometry	100 mm KNO_3_	ML	13.1	13.1
TPEN	60	potentiometry	100 mm KNO_3_	ML	16.4	16.4
DTBA	39	potentiometry	100 mm KNO_3_	ML, ML_2_, M_2_L_3_	n.c.	12.8
DTT	38	potentiometry	100 mm KNO_3_	ML, ML_2_	n.c.	10.4
Ac‐CC‐NH_2_	63	potentiometry	100 mm KCl	ML_2_	n.c.	12.6
Ac‐YCSSCY	62	potentiometry	100 mm NaClO_4_	ML, ML_2_	n.c.	14.8
Ac‐EEGCCHGHHE‐NH_2_	63	potentiometry	100 mm KCl	ML, ML_2_	n.c.	12.5
γECγEC (PC2)	83	potentiometry	100 mm KNO_3_	ML, ML_2_	n.c.	10.7
XPA zf	23	UV/Vis RT	50 mm phosphate, pH 7.4	ML	12.8	12.8
CP1 zf (CCCC)	61	UV/Vis RT	100 mm HEPES, 50 mm NaCl, pH 7.0	ML	13.4	13.4
CP1 zf (CCCH)	61	UV/Vis RT	100 mm HEPES, 50 mm NaCl, pH 7.0	ML	11.2	11.2
CP1 zf (CCHH)	61	UV/Vis RT	100 mm HEPES, 50 mm NaCl, pH 7.0	ML	8.7	8.7
TT‐2D zf	84	UV/Vis pHT	200 mm HEPES, 100 mm NaCl, pH 7.5	ML	8.5	8.5
MT2	59	UV/Vis pHT	5 mm Tris‐HCl, 50 mm NaCl, pH 7.0	M_7_L	14.4	14.4
					15.8	15.8
CmtR	58	CC	10 mm Bis‐Tris, 400 mm NaCl, pH 7.0	ML	12.2	12.2
CadC	57	CC	5 mm MES, 200 mm NaCl, pH 7.0	ML	12.6	12.6

[a] DTBA=dithiobutanoic acid, DTT=dl‐dithiothreitol, EDTA=ethylenediaminetetraacetic acid, HEDTA=*N‐(*2‐hydroxyethyl)ethylenediamine‐*N*,*N′*,*N′*‐triacetic acid, TPEN=*N*,*N*,*N*′,*N*′‐tetrakis(2‐pyridylmethyl)ethylenediamine, Bis‐Tris=2,2‐bi(hydroxymethyl)‐2,2′,2′′‐nitrilotriethanol, MES=2‐(*N*‐morpholino)ethanesulfonic acid.

### Zn^II^‐to‐Cd^II^ swap in zinc hook domain

**Figure 8:**
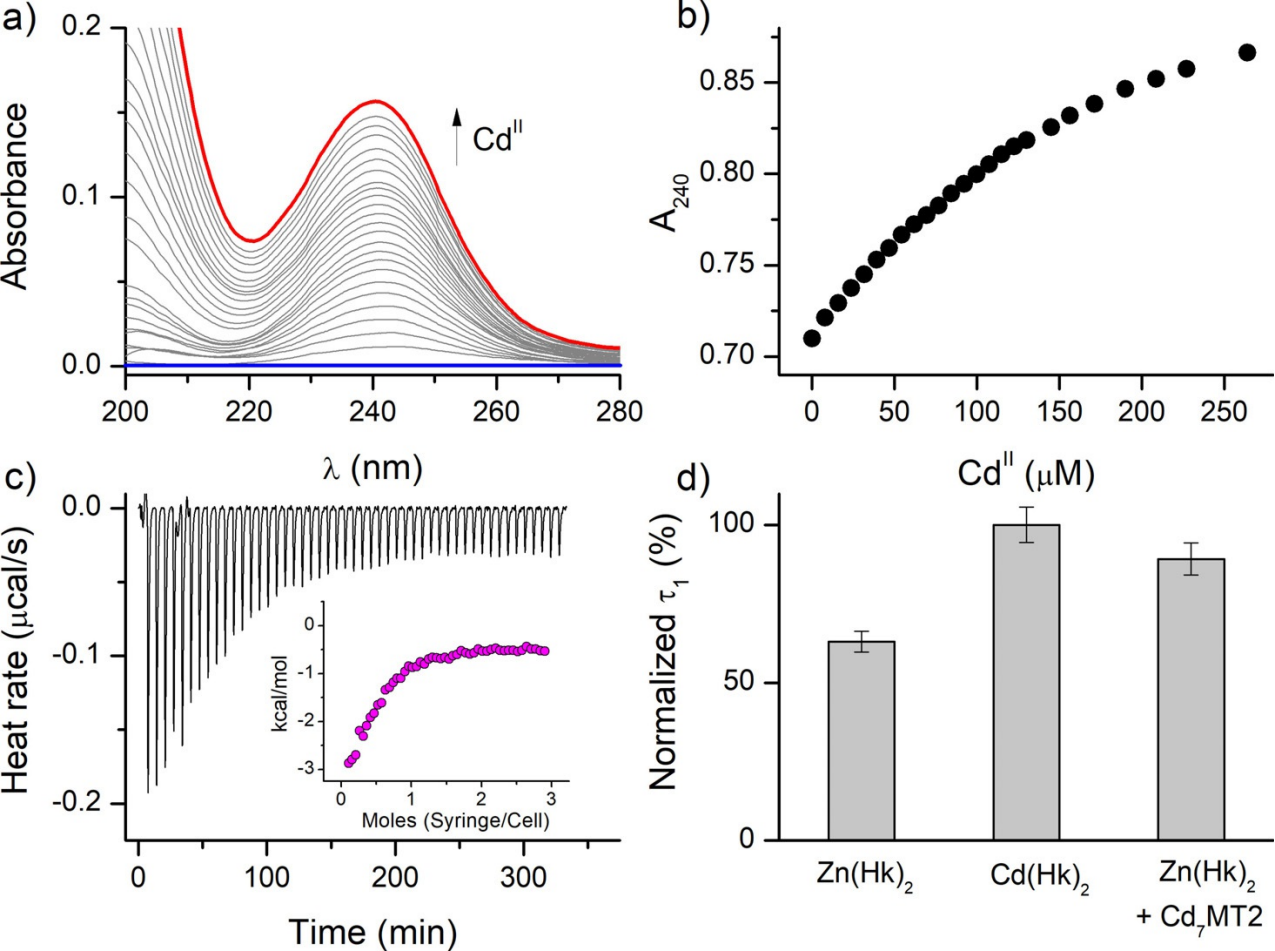
Zn^II^‐to‐Cd^II^ swap in hook peptides illustrated by UV spectroscopy, ITC, and anisotropy decay analysis. a) Differential UV/Vis spectra of 100 μm Hk14 and 500 μm Zn^II^ titrated with Cd^II^. b) Absorbance changes at 240 nm from a) illustrated as a function of increasing Cd^II^ concentration indicating metal‐ion exchange. c) ITC analysis of 100 μm Hk14 with 250 μm Zn^II^ stepwise titrated with 1000 μm Cd^II^, represented as a baseline‐subtracted heat rate as a function of time. The inset shows heat per mole of titrant as a function of the Cd^II^/Zn(Hk14)_2_ molar ratio. d) Anisotropy decay analysis of 500 nm FAM‐Hk45 presented as rotational correlation time changes normalized to the apo form (0) and Cd(Hk45)_2_ of Zn(Hk)_2_, Cd(Hk)_2_, and Zn(Hk)_2_ with equimolar Cd_7_MT2. Error bars represent standard deviations from averaging four consecutive samples.

Data presented above show that Cd^II^ complexes of the minimal and elongated hook domain are significantly more stable than Zn^II^ counterparts and other protein, peptide, and low‐molecular‐weight organic complexes. This should be reflected by an efficient Zn^II^ swap when Cd^II^ ions are added to the zinc hook domain. To examine experimentally how fast and efficient the substitution of metal ions occurs, we titrated the Zn^II^ complex of Hk14 and Hk45 with Cd^II^ and observed changes occurring in UV/Vis and CD spectra. This was possible due to the formation of energetically lower LMCT bands at 230–250 nm for Cd^II^ in comparison with those of Zn^II^ complexes.^37^, ^38^, ^39^ This effect has also been observed in CD spectra by bathochromic shift of ellipticity negative maxima and their intensity increase when Cd^II^ swap occurred. To measure the equilibrated states of the reaction during titration properly, the kinetics of Zn^II^‐to‐Cd^II^ swap was examined first. Figure S6 (Supporting Information) indicates that in the case of both Zn(Hk14)_2_ and Zn(Hk45)_2_ complexes the time necessary for metal‐ion‐exchange completion is lower than 2 minutes, indicating rapid kinetics of the metal swap. Exchange for Hk14 was shown to occur under first‐order kinetics with a rate constant calculated to be about 0.015 s^−1^, and the rate for Hk45 is even faster and was impossible to determine under standard settings. When the Zn(Hk14)_2_ complex was titrated with Cd^II^, the metal exchange observed by an increase in the LMCT band intensity (Figure 8 a) reveals almost direct exchange, indicating that the reaction is not only rapid but also highly efficient, as expected, due to the difference in stability constants of Cd^II^ and Zn^II^ complexes. Fivefold molar excess of Zn^II^ over Hk14 slows down the exchange in such a way that a higher concentration of Cd^II^ is necessary to swap the hook‐bound Zn^II^. This enables calculation of the formation constant *K*_12_ based on the fixed constant of Zn(Hk14)_2_ and total Cd^II^ concentration (Figure 8 b).^54^ The *K*_12_ values calculated based on the exchange mode are slightly higher than the values obtained potentiometrically or in competition with complexones (Table 2), probably due to intrinsically low resolution of the method in terms of stability determination^47^, ^53^ and were not included in further discussion. Nonetheless, the results prove that Zn^II^‐to‐Cd^II^ swap is a fast and efficient process that can occur inside a cell, causing Cd^II^‐induced toxic effects.

To illustrate the Zn^II^‐to‐Cd^II^ exchange event located inside Rad50’s hook domain, we performed isothermal titration calorimetry (ITC) analysis in a manner similar to the previous approach. 100 μm Hk14 with 2.5‐fold molar excess of Zn^II^ was titrated stepwise with 500 μm Cd^II^. As shown in Figure 8 c, Cd^II^ readily displaces the hook‐bound Zn^II^ ion, which is represented as a slowly decreasing exothermic process (inset) of a tendency much like the one from the UV spectroscopy experiment (Figure 8 b). Thermodynamics associated with the metal exchange reaction is discussed separately below.

Living organisms possess multiple factors that guard cells from Cd^II^ toxicity, with the two most important examples being glutathione (GSH) and metallothioneins. Regardless of the fact that GSH is present in the cytoplasm in millimolar levels, Cd^II^ maintains the capacity to infiltrate protein targets. It is a known fact that Cd^II^ accumulation in mammals occurs in soft organs, especially in the liver and kidneys.^64^, ^65^, ^66^ Metallothionein, found originally as a Cd^II^‐binding protein in the horse kidney cortex, binds this metal ion very tightly in two distinct clusters: four Cd^II^ ions in the α‐cluster and three Cd^II^ ions in the β‐cluster. However, isolated hepatic metallothionein from cadmium‐toxicated mammals demonstrates a heterogeneous nature.^67^ Metallothionein binds at the same time Cd^II^ as well as Zn^II^ ions at various molar ratios; however, only a Cd_5_Zn_2_MT species was characterized structurally, and the crystal structure of this species is the only X‐ray structure of mammalian MTs.^68^, ^69^ In this study, by using Cd^II^‐reconstituted metallothionein‐2, we aimed to examine whether tightly bound Cd^II^ ions by this protein can be transferred to the zinc hook domain of the Rad50 protein, which as a consequence could shed a light on Cd^II^ ion distribution in cells.

It is worth mentioning that seven Cd^II^ ions are bound in MT2 with two distinct affinities: three ions corresponding to the β‐cluster with averaged log *K*=14.4 and four bound in the α‐cluster with log *K*=15.8, which is in contrast to that of zinc MT2.^58^, ^70^, ^71^ Stability data obtained in this study suggest that Cd^II^ could be easily transferred from human Cd_7_MT2 to the hook domain. To examine Cd^II^ transfer from MT2, which demonstrates strong absorption in the UV range due to LMCT bands occurring between the Cys residue and Cd^II^, we used the fluorescently labeled Hk45 peptide and monitored anisotropy changes associated with this process. Our study showed that anisotropy decay of Zn^II^ and Cd^II^ hook complexes differs in terms of rotational correlation time values, which enables its use for type‐detection of metal ions bound to peptides.

In the first step of metal swap, similarly to free Cd^II^ ion substitution, we examined the kinetics of the transfer. Fluorescein emission changes presented in Figure S7 (Supporting Information) indicate that the kinetics is very fast and that the reaction occurs completely in the time window below 1 minute. Kinetics of the swap seem to be equally fast for Cd^II^ alone (i.e., not bound to MT2) and fully Cd^II^‐metalated MT2. To show that both act in a similar fashion, we examined Zn(Hk45)_2_ and Cd(Hk45)_2_ with either addition of Cd^II^ or Cd_7_MT2a. The control sample's rotational correlation time, being in this particular case of Cd(Hk45)_2_, was similar after separate addition of Cd^II^ and Cd_7_MT2a in 0.5 and one molar equivalents, respectively (Figure S8, Supporting Information). However, for the Zn(Hk)_2_ sample the parameter changed towards Cd^II^‐specific values, both for Cd^II^ and Cd_7_MT2a additions (Figure 8 d). Overall, the results show that Zn^II^‐to‐Cd^II^ swap occurs even with the additional Cd^II^‐chelating component, and we suggest that it may be a relevant cellular process where MT is one of the major barriers against Cd^II^ toxicity.

### Structure determination of Zn^II^‐ and Cd^II^‐substituted hook domain

All spectroscopic and thermodynamic studies obtained in this report demonstrate that the minimal fragment of the hook domain (Hk14) forms a well‐defined and highly stable structure as a complex with Cd^II^ ions. Stability constants obtained for longer models show that sequence elongation does not affect the stability of the complex significantly, suggesting that folding of the hook domain starts from the short 14‐amino‐acid‐long fragment responsible for Rad50 dimerization. Although a *P. furiosus* Rad50 zinc hook domain's crystal structure was solved more than two decades ago, we are still missing a structure of a Zn^II^‐loaded complex because the solved one was actually a mercury hook (Hg(Hk112)_2_, PDB code: 1L8D).^26^ In light of the results presented above, we speculate that the zinc hook may be in fact structurally different in solution, given that the Cd^II^‐loaded hook seems to possess a different dimeric arrangement. Thus, we aimed to determine structures of this motif to provide a new look at this alluring interprotein site. For this reason, we obtained 5 mm Zn^II^ and Cd^II^ complexes of the Hk14 hook model and performed a number of one‐ and two‐dimensional NMR spectroscopic experiments, including ^1^H–^13^C HSQC, TOCSY, and NOESY, obtaining 130 and 190 NOE peaks for Zn^II^ and Cd^II^, respectively. From these, 20 and 15 peaks for Zn^II^ and Cd^II^ complexes, respectively, were rejected at the initial assignment.

**Figure 9:**
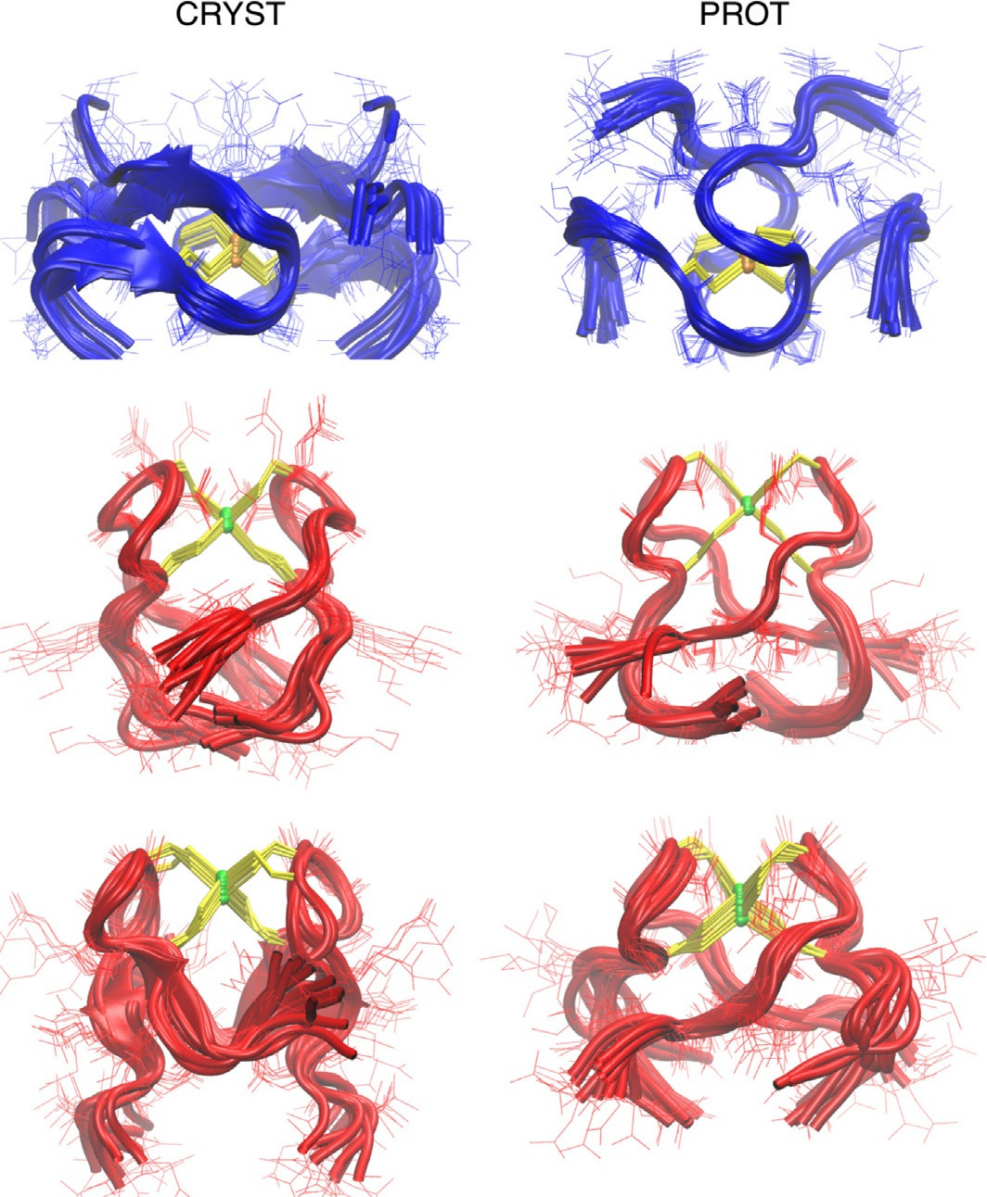
Ensembles of ten dimeric structures of the Hk14 peptide with Zn^II^ (blue chains) and Cd^II^ (red chains). Structures in the left column were determined with the CRYST procedure, and structures in the right column were determined with the PROT procedure (see the Supporting Information). Cysteine side chains are shown in yellow stick representation, and Zn^II^ and Cd^II^ are shown as orange and green spheres, respectively. The relative arrangement of loops of two analogous complexes with different ions differs significantly (see Results and Discussion). Two structures of complexes with Cd^II^ (middle and lower rows) correspond to two sets of chemical shifts obtained from the NMR spectroscopic experiments. In the computation procedure for the bottom left structure, two weak N−S hydrogen‐bond restraints were removed.

The ten lowest‐energy structures of Zn(Hk14)_2_ and Cd(Hk14)_2_ obtained with PROT and CRYST procedures (with hydrogen‐bond restraints obtained from protection factors and crystal structures, respectively, as explained in the Supporting Information) are shown in Figure 9. The statistics of violated restraints are collected in Table S5. The energy of violated restraints is relatively low for both Zn(Hk14)_2_ and the first conformation of Cd(Hk14)_2_. For the second conformation of Cd(Hk14)_2_ computed with CRYST restraints, significant (>0.5 Å) violations of two hydrogen bonds (Val7 N–Cys5 S and Arg10 N–Cys8 S) were observed in 3 out of 10 structures, leading to a substantial rise in the average energy of restraints. Therefore, we ran additional calculations without restraints put on these two weak hydrogen bonds and obtained structures with reasonable mean energy of NOE and hydrogen‐bond restraints (see Table S5). Calculations with PROT restraints led to structures with very small violations of H‐bond restraints, but violations of NOE restraints increased in comparison to the structures obtained with CRYST restraints; although only 2 out of 10 computed structures have severely violated NOE restraints (NOE energy close to 200 kcal mol^−1^). Nevertheless, structures obtained for the second set of chemical shifts are similar to those obtained for the first set, as can be seen in Figure 9.

The structures of peptides with Cd^II^ and Zn^II^ have clearly different arrangements of chain termini (Figure 9 and Figure S9, Supporting Information). The N‐ and C‐termini of both Hk protomers bound to Cd^II^ are located close to each other (red structures in middle and lower panels of Figure 9), whereas the termini of the same peptides bound to Zn^II^ are located on the opposite sides of the complex (blue structures in upper panel of Figure 9 and Figure S9, Supporting Information). Described structural differences may also be present in complexes of the investigated ions with longer Hk peptides, leading to different arrangements of helices forming coiled coils and a global structural change of the entire MR(N/X) complex. Nonetheless, further structural studies on longer protein fragments need to be undertaken to confirm this hypothesis.

### Molecular bases of elevated stability

Data reported herein clearly show that the Rad50 hook complex with Cd^II^ is significantly more stable, by around two orders of magnitude in terms of *K*_12_, compared with its physiological counterpart that is Zn^II^. Moreover, having established the Cd^II^‐binding affinity of the central fragment of *P. furiosus* Rad50, we thereby documented the most stable Cd^II^–peptide complex analyzed so far (Table 3). Such extreme stability is likely to be generated by multiple molecular factors, including enthalpic and entropic contributions from Cd−S bond formation and metal‐induced nucleation, to name a few. Figure 6 a and Table S6 (Supporting Information) show that formation constant *K*_12_ values of Cd(Hk)_2_ complexes are almost proportionally shifted towards higher stabilities than those of Zn^II^ hook model complexes. This indicates that these two types of complexes behave similarly when the hook motif is elongated from both ends. In Figure 6 a, blue and red arrows indicate two events of Gibbs free energy contribution for Zn^II^ and Cd^II^ complexation, respectively, demonstrated as an energy difference (ΔΔ*G*°_Cd/Zn_) as a function of peptide length. The first event occurs between 4 and 14 amino acid residues in the hook motif, and the second event (less pronounced) occurs between 23 and 45, which correspond to the metal‐binding‐induced formation of the β‐hairpin and nucleation of the coiled‐coil region, respectively.^33^ The difference between formation constants (log K12CdII −log K12ZnII ) is similar for Hk10, Hk14, Hk37, Hk45, and Hk130 and is 1.98±0.04, which corresponds to −2.7 kcal mol^−1^ of Gibbs free energy and, with a high level of confidence, is correlated with increased contribution of bond formation enthalpy for Cd−S compared with that for Zn−S (Table S6, Supporting Information). However, the log *K*_12_ differences presented in Figure 6 b demonstrate the presence of two regions where Cd^II^ complexes are even more stabilized than the rest of the length‐differentiated peptide series. The first extra‐elevated region occurs for hook peptides between Hk4 and Hk8 with a maximum at Hk6. This significant stability increase is very likely connected to differences in the ionic radii of both ions and the possibility of generating alternate torsion angles of peptide bonds or even diverse intra‐ and intermolecular connections.^33^ Another, less pronounced, region indicating an additional difference between the two metal‐ion complexes is present at the amino‐acid length of 31. This region is more complicated to explain, but we suggest it may be caused by a shift in the coiled‐coil nucleation event towards shorter hook lengths for Cd(Hk)_2_, which is somewhat supported by the CD data showing higher capacity for the hook's helical nucleation of Cd^II^ compared with that of Zn^II^, judged by qualitative evaluation (Figure S10, Supporting Information).

**Figure 10:**
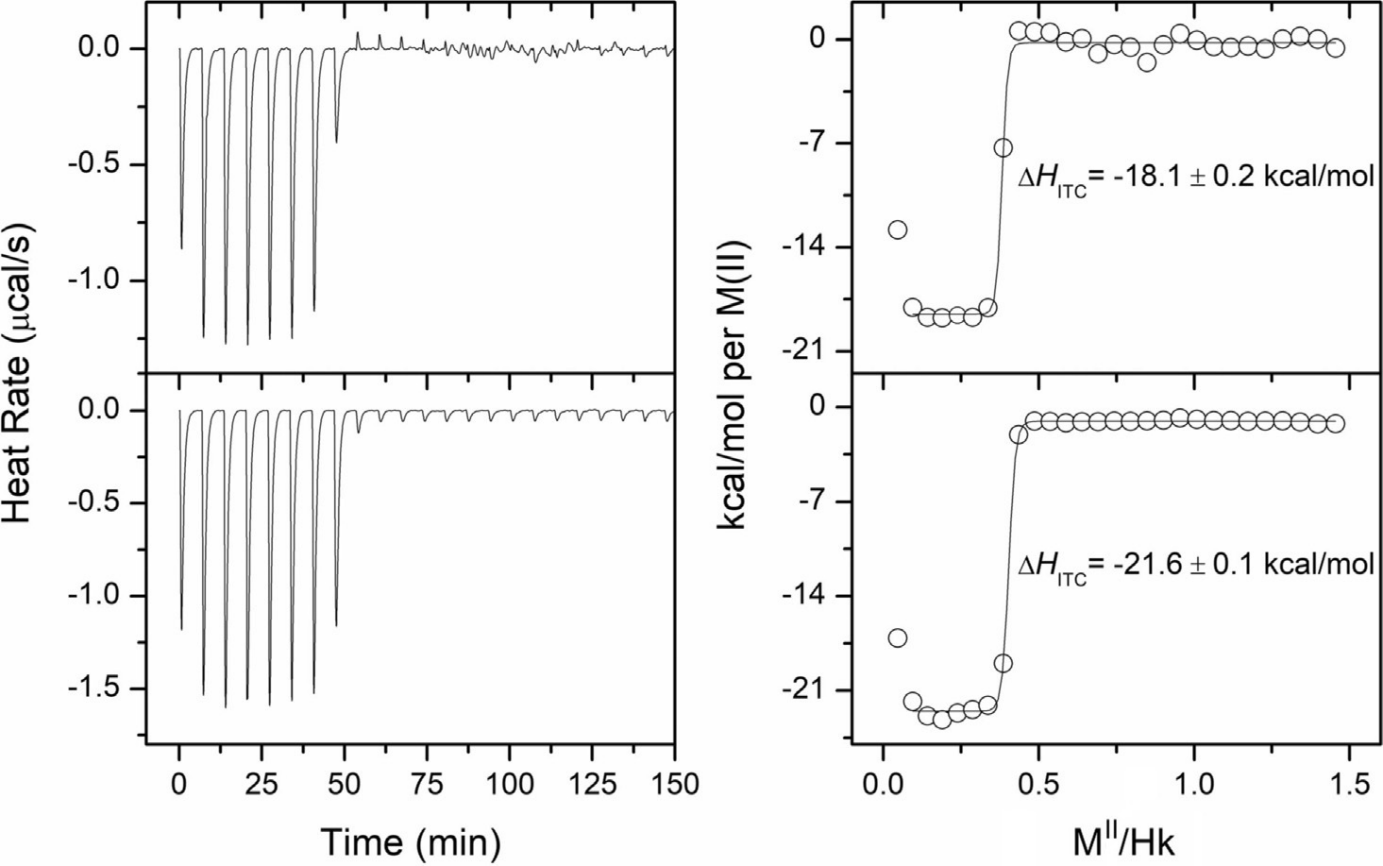
Isothermal titration calorimetry analysis of 100 μm Hk14 stepwise titrated with 650 μm ZnSO_4_ (upper panel) or CdSO_4_ (lower panel). Left graphs illustrate baseline‐subtracted heat rate over time, and right graphs show observed enthalpy per mole of titrant (Zn^II^ or Cd^II^) as a function of molar M^II^‐to‐Hk14 ratio.

To assess what thermodynamic processes are involved and how they affect the stability of Cd(Hk)_2_, we performed ITC analysis of apo Hk4–45 titrated with Cd^II^ and Zn^II^ (Figures S11, S12, Supporting Information) to illustrate differences between complexation of both metal ions as well as the Zn^II^ complex titrated with Cd^II^ to visualize the energetic outcome of the Zn^II^‐to‐Cd^II^ swapping phenomenon (Figure 8 c). Figure 8 c shows that Cd^II^ efficiently replaces Zn^II^ inside the hook motif, even in excessive Zn^II^ conditions. Moreover, it proves the existence of a favorable enthalpic contribution of the swapping event, which seems to be approximately −2.6 kcal mol^−1^, a value that corresponds well with the −2.7 kcal mol^−1^ value obtained from potentiometric and spectroscopic studies (Figure 6 b and Table S6, Supporting Information). On the other hand, independent experiments with Zn^II^ and Cd^II^ titrations of apo peptides suggest that complex formation enthalpies of Zn(Hk14)_2_ and Cd(Hk14)_2_ are −18.1 and −21.6 kcal mol^−1^, respectively, which gives −3.5 kcal mol^−1^ for ΔHCdIIITC −ΔHZnIIITC (Figure 10). The 0.9 kcal mol^−1^ gap between these two values (the Zn^II^‐to‐Cd^II^ swap enthalpy subtracted from the apo‐to‐holo metalation enthalpy difference) can be explained by the contribution of the folding enthalpy (ΔHfold∘ ), which our results prove to be different for Zn^II^ and Cd^II^ complexes with Rad50.^72^, ^73^, ^74^ During the swap event, the hook is already present as a folded dimer, and the energetic contribution of metal‐ion‐exchange‐induced structural changes is too small to be observed directly through this type of ITC experiment; hence, the observable difference is mainly dictated by the enthalpy of the Cd−S bond formation. On the other hand, direct titration of the apo peptide with Zn^II^ or Cd^II^ includes contributions of M−S bond formation (${\Delta H}_{M-S}^{{}^{\circ }})$ , Cys thiol group deprotonation (${n_{H}\Delta H}_{CysH}^{{}^{\circ }})$ , protonation of HEPES (${n_{H}\Delta H}_{HEPES}^{{}^{\circ }}$ ), and protomer folding (${\Delta H}_{fold}^{{}^{\circ }})$ [Eqs. (3), (4)].

(3) $${\Delta H}^{ITC}={\Delta H}^{{}^{\circ }}+{n_{H}\Delta H}_{HEPES}^{{}^{\circ }}$$

(4) $${\Delta H}^{{}^{\circ }}=\;{\Delta H}_{M-S}^{{}^{\circ }}+{\Delta H}_{fold}^{{}^{\circ }}+{n_{H}\Delta H}_{CysH}^{{}^{\circ }}$$

Assuming that Cys deprotonation and HEPES protonation enthalpies are equal for both Zn^II^ and Cd^II^ titrations, the energetic outcomes of metal–sulfur bond formation and protomer folding have to be different.^75^ Taking the above into account, one may conclude that the energetic cost of Rad50 protomer folding and dimerization is in fact different for Zn^II^ and Cd^II^ and can be roughly estimated for Cd(Hk14)_2_ by the following subtraction: ${\Delta \Delta H}_{{\mathrm{Cd}}^{\mathrm{II}}-{\mathrm{Zn}}^{\mathrm{II}}}^{ITC}-{\Delta H}_{swap}^{ITC}$ , which equals −0.9 kcal mol^−1^. Moreover, the entropic contribution, derived from the Gibbs equation as *T*Δ*S*°, is slightly lower for Cd(Hk14)_2_ than for Zn(Hk14)_2_, with 23.13 and 23.94 kcal mol^−1^, respectively, suggesting that Cd^II^ may induce alternative hook folding that manifests as an entropy loss (Figure S13, Supporting Information). These calculations demonstrate that the molecular basis of the Cd^II^ complexes’ elevated stability originates from the higher enthalpic contribution of both Cd−S bond formation and Cd^II^‐induced protomer folding, compared with Zn^II^ complexes, therefore indicating that Zn(Hk)_2_ and Cd(Hk)_2_ present different structural arrangements.

To evaluate values of thermodynamic parameters obtained in this study, we aimed to compare them with published ITC data for 4Cys metal‐binding sites. Although the literature lacks consistent thermodynamic data for this particular metal‐binding site, as well as the Cys residue alone binding to Zn^II^ and Cd^II^ ions, we managed to find some values. Krizek et al. obtained ΔGCdII∘ =−1.9 kcal mol^−1^ for consensus zinc finger CP1 with the 4Cys metal‐binding motif.^61^ Another zinc finger with a 4Cys binding motif, XPA, showed lower Gibbs free energy of around −4 kcal mol^−1^ for Cd^II^ complexation.^23^ On the other hand, Zn_4_MT3 from *Musa acuminata* titrated with Cd^II^ gave observable heats of around −3 kcal mol^−1^.^76^ Although the presented values seem to differ quite significantly, they are still comparable to our data, especially taking into account that none of the experiments were performed in exactly the same way, nor did they maintain high analytical standards. Nonetheless, the difference in Gibbs free energy values of Cd^II^ and Zn^II^ complexation by the Rad50 hook domain or other 4Cys binding motifs seems to vary at around −3 kcal mol^−1^.

### Biological significance

The Rad50 protein is an integral constituent of the MR(N/X) complex present in every living organism analyzed so far. MR as a heterotetrameric (or heterohexameric MR(N/X) in higher organisms) complex, comprised of two units of Mre11 nuclease and two units of Rad50, guards genomic integrity by sensing and repairing double‐stranded DNA breaks that otherwise left unrepaired would cause deleterious genotoxic effects inevitably leading to cell death.^27^ Given that Mre11 is responsible for processing of the broken DNA ends, Rad50 plays a more structural role, acting as a lengthy scaffold for the complex and a major dimerization factor through action of the zinc hook domain located at the apex of the MR(N)_._ complex. It is believed that the MR(N/X) complex is a flexible one with highly dynamic properties that enable it to adopt multiple conformational assemblies.^26^, ^28^, ^29^, ^31^, ^77^ Recent findings indicate that conformation‐altering signals can be generated both at Rad50’s ATPase globular and zinc hook domain apexes and transferred through an immensely long coiled‐coil segment that spans 500 Å.^34^, ^35^, ^43^, ^45^, ^77^, ^78^, ^79^ At this point, two conformational states of the MR(N/X) complex seem to play a major functional role: closed conformation that binds tightly to DNA substrates and open conformation that enables DNA processing and repair.^34^, ^80^ Both assemblies interact differently with DDR proteins and seem to be preferentially activated during different phases of the cell cycle.^81^, ^82^ These data indicate that the zinc hook domain is much more than a simple dimerization motif and could possibly regulate the functional status of the entire complex. We have previously shown that any kind of amino‐acid‐substitution‐based alterations render the zinc hook abrogated, destabilizing Zn^II^ binding in vitro and DNA damage repair in vivo.^33^, ^43^ In this sense, any factor that inflicts structural changes upon the zinc hook domain has to be treated as a potential genotoxic agent.

Taking the above into account, Cd^II^ represents an evident threat to Rad50’s functional status and therefore a threat to genomic integrity. Considering that Zn^II^‐binding proteins represent approximately 10 % of the cell's proteome, Cd^II^‐swapping outcomes could be deleterious for multiple biochemical reasons. Intuitively, the most detrimental toxic effect pertains to DNA‐binding proteins and transcription factors that ubiquitously harbor Zn^II^‐rich domains. Therefore, Rad50, with its zinc hook domain, poses as a perfect target for Cd^II^ toxicity.

Experimental results presented herein prove that Cd^II^ bound to Rad50 alters its structural status. The Cd(Rad50)_2_ complex presents an augmented molecular volume that coincides with formation of extended helical regions, compared with Zn(Rad50)_2_. Furthermore, the Cd^II^ ion is bound so tightly that it renders the zinc hook domain effectively blocked in one rigid conformation and incapable of any Zn^II^‐ or DDR‐protein‐driven structural rearrangements that seem to be crucial for the MR(N/X) complex's multifunctionality.

## Conclusion

We hereby documented the most stable Cd^II^–peptide complex described so far, with sub‐zeptomolar affinity as a Cd(Rad50)_2_ dimer, and determined molecular bases of Cd^II^‐induced stability elevation compared with its counterpart that naturally allocates in the Rad50 hook domain, that is, Zn^II^. Cd^II^, although similar to Zn^II^ in terms of physical properties, induces significant structural changes in the Rad50 hook domain. The reported increase in rotational correlation time and altered NMR spectroscopic structure suggest the possibility of Cd^II^‐binding‐induced global MR(N/X) complex rearrangement, implying that this metal ion is capable of abrogating DNA‐damage sensing and repair functions of the MR(N/X) complex in a cellular context. Additionally, the approximately two‐orders‐of‐magnitude increase in Cd(Rad50)_2_ stability compared with that of Zn(Rad50)_2_ supports the notion that Cd^II^ is bound almost irreversibly to its target and renders it incapable of Zn^II^‐ and DDR‐factor‐governed flexibility that seems to be crucial for proper double‐stranded DNA damage repair. Our results show that this particular interaction of Cd^II^ may be one of the major reasons for its still‐unresolved genotoxicity and show how Cd^II^ ions can impact intermolecular zinc‐binding sites in oligomeric proteins.

## Experimental Section

### Materials

*N*,*N*‐Diisopropylethylamine (DIEA), 9‐fluorenylmethoxycarbonyl (Fmoc)‐protected amino acids (Fmoc‐Ala‐OH**⋅**H_2_O, Fmoc‐Arg(Pbf)‐OH, Fmoc‐Asn(Trt)‐OH, Fmoc‐Asp(O*t*Bu)‐OH, Fmoc‐Cys(Trt)‐OH, Fmoc‐Gln(Trt)‐OH, Fmoc‐Gln(Trt)‐OH, Fmoc‐Glu(O*t*Bu)‐OH, Fmoc‐Gly‐OH, Fmoc‐His(Trt)‐OH, Fmoc‐Ile‐OH, Fmoc‐Leu‐OH, Fmoc‐Lys(Boc)‐OH, Fmoc‐Met‐OH, Fmoc‐Phe‐OH, Fmoc‐Pro‐OH, Fmoc‐Ser(*t*Bu)‐OH, Fmoc‐Thr(*t*Bu)‐OH, Fmoc‐Tyr(*t*Bu)‐OH, Fmoc‐Val‐OH), piperidine, *O*‐(benzotriazol‐1‐yl)‐*N*,*N*,*N′,N′*‐tetramethyluronium hexafluorophosphate (HBTU), and dl‐dithiothreitol (DTT) were purchased from Iris Biotech GmbH. Trifluoroacetic acid (TFA), 1,2‐ethanedithiol (EDT), thioanisole, anisole, triisopropylsilane (TIPS), COMU, guanidine hydrochloride (GdnHCl), 4‐mercaptophenylacetic acid, tris(2‐carboxyethyl)phosphine hydrochloride (TCEP), ethylenediaminetetraacetic acid (EDTA), HCl (trace metal grade), bis(β‐aminoethyl ether)‐*N*,*N*,*N*′*,N*′‐tetraacetic acid (EGTA), *N*‐carboxymethyl‐*N′*‐(2‐hydroxyethyl)‐*N*,*N′*‐ethylenediglycine trisodium salt (Na_3_‐HEDTA), NaClO_4_ **⋅**H_2_O, ZnSO_4_ **⋅**7 H_2_O, and CdSO_4_ **⋅**8/3 H_2_O were from Merck KGaA. Diethyl ether, acetic anhydride, dichloromethane (DCM), and NaCl were purchased from Avantor Performance Materials Poland S.A. Chelex 100 resin was from Bio‐Rad, 4‐(2‐hydroxyethyl)piperazine‐1‐ethanesulfonic acid sodium salt (HEPES) was from Bioshop, 5,5′‐dithiobis(2‐nitrobenzoic acid) (DTNB) was from TCI Europe N.V., TentaGel R RAM and TentaGel S‐NH_2_ resins were from Rapp Polymere GmbH, and dimethylformamide (DMF) and acetonitrile (MeCN) were from VWR. All of the experiments were performed in chelexed buffers and solutions. All buffers were prepared with Milli‐Q water obtained with a deionizing water system (Merck KGaA).

### Peptide synthesis

Zinc hook peptides (Hk peptides) were synthesized by solid‐phase peptide synthesis (SPPS) using an Fmoc‐strategy on a TentaGel R RAM Amide Rink (Rapp Polymere GmbH, Tübingen, Germany) resin (substitution 0.2 mmol g^−1^) and a Liberty 1 microwave‐assisted synthesizer (CEM) as described previously.^33^, ^85^ Peptides were N‐terminally acetylated with acetic anhydride or fluorescently modified with a dansyl (Dns) moiety or 5(6)‐carboxyfluorescein (FAM )derivatives. Cleaved peptides were precipitated and washed with cold diethyl ether and purified on a C18 column (Phenomenex) with a gradient of acetonitrile and 0.1 % TFA using a Dionex Ultimate 3000 HPLC system. The identity of peptides was confirmed with an API 2000 Applied Biosystems ESI‐MS instrument. Concentration of peptide stocks in 10 mm HCl was determined using a sulfhydryl‐group reactant, DTNB (*ϵ*=14,150 m ^−1^ cm^−1^ at 412 nm), prior to each experiment.^86^

### Expression and purification of metal‐free Hk130 and human metallothionein‐2

The production of *P. furiosus* Hk130 and human metallothionein‐2 (MT2) relied on a previously established protocol using the pTYB21 expression vector (IMPACT Protein Purification System, NEB) and *Escherichia coli* (*E. coli*) BL21‐CodonPlus (DE3)‐RIL strain.^71^ The expression vector is deposited in the Addgene plasmid repository (https://www.addgene.org), plasmid ID 105693 (MT2a). Transformed cells were cultivated in 4 L of LB or TB medium, respectively, and grown at 37 °C until OD_600_ was 0.4–0.5, and then induced with 0.1 mm IPTG. Cultures were incubated overnight at 20 °C with vigorous shaking and subsequently collected by centrifugation at 4500×*g* for 20 min at 4 °C. The pellets were resuspended in ice‐cold buffer A (20 mm HEPES, pH 8.0, 500 mm NaCl, 1 mm EDTA, 1 mm TCEP) and lysed by sonication on ice for 30 min, followed by centrifugation at 20 000×*g* for 15 min. Clarified cell extracts were incubated overnight with chitin resin at 4 °C with mild shaking. After the incubation, the resin was washed with 20 bed volumes of buffer A with increased salt concentration (1 m NaCl) to reduce nonspecific binding of other *E. coli* proteins. To induce the cleavage reaction, 25 mL of buffer B (20 mm HEPES, pH 8.0, 500 mm NaCl, 1 mm EDTA, 100 mm DTT) was added to the resin, and the mixture was incubated for 36–48 h at room temperature with mild shaking. Eluted protein solutions were acidified to pH≈2.5–3.0 with 7 % HCl and concentrated to a small volume using Amicon Ultra‐4 Centrifugal Filter Units with NMWL of 3 kDa (Merck Millipore, USA). Hk130 protein was purified by reverse‐phase HPLC in a 0.1 % TFA/acetonitrile gradient (Dionex) followed by lyophilization, and MT2 was purified on an SEC‐70 gel filtration column (Bio‐Rad, USA) equilibrated with 5 mm HCl.^70^ The identity of metal‐free MT2 and Hk130 protein was confirmed by ESI‐MS, using an API 2000 instrument (Applied Biosystems, USA); the average molecular weight (MW) calculated was 6042.0/6042.2 Da (calcd/exp) and 15217.6/15217.8 Da (calcd/exp), respectively.

### Reconstitution of cadmium metallothionein‐2

To reconstitute the protein with Cd^II^, aliquots of thionein in 5 mm HCl were mixed with cadmium sulfate at a molar ratio of 1:9 under a nitrogen blanket, and the pH adjusted to 8.6 with 1 m solution of Tris base and purified on an SEC‐70 gel filtration column equilibrated with 20 mm Tris‐HCl buffer, pH 8.6. The concentration of the purified protein was obtained spectrophotometrically, with DTNB and PAR assays for the thiol and Cd^II^ concentration, respectively.^86^, ^87^ Additionally, samples were analyzed by inductively coupled plasma (ICP) analysis (ICP‐AES iCAP 7400, Thermo Scientific) to confirm the spectrophotometric results.

### Spectroscopic studies

The binding properties of hook peptides to Cd^II^ were examined by electronic absorption spectroscopy and circular dichroism by using a Cary 300 spectrophotometer (Varian) and Jasco J‐1500 spectropolarimeter (Jasco) with a Peltier heating/cooling system, respectively. Spectra were recorded at 25 °C in the wavelength range of 200–280 nm to observe LMCT transitions^39^, ^85^ as well as secondary structure changes.^33^ Three accumulations were averaged using a 5 nm band width, a 200 nm min^−1^ scanning speed, and a 1.0 nm data pitch. Depending on Hk length, 25–100 μm peptide in 20 mm Tris‐HCl buffer (pH 7.4, *I*=0.1 m from NaClO_4_) was titrated with small aliquots of concentrated CdSO_4_ to achieve molar ratios from 0 to 2 over Hk. After each addition of Cd^II^, samples were equilibrated for 2–5 min and the spectra were recorded. All measurements were performed in the presence of fourfold excess of TCEP over Cys residue to avoid their oxidation. TCEP forms a very weak Cd^II^ complex (log *K* ^7.4^=3.3) compared with Hk peptides, and its metal‐binding ability can be neglected.^36^ Zn^II^‐to‐Cd^II^ replacement experiments were conducted on either equimolar or fivefold excess (to slow down the exchange) of Zn^II^ molar equivalent over Hk with Cd^II^ titration as described previously.

### Potentiometry

The protonation constants of the Hk4–Hk14 zinc hook peptides and stability constants of their Cd^II^ complexes were determined at 25 °C at 0.1 m ionic strength (from KNO_3_) by pH‐metric titration over a range of 2.5 to 10.8, using a Molspin automatic titrator under argon with standardized 0.1 m NaOH as a titrant. The data were analyzed using SUPERQUAD software.^49^

### Fluorimetry and fluorescence anisotropy study

Fluorimetric studies were conducted with a Jobin Yvon Fluoromax‐3 spectrofluorimeter (Horiba) equipped with a Peltier‐thermostatted cell holder. Competition intra‐FRET analysis was carried out as follows: 1 mm concentration of the appropriate Zn^II^ chelator (TPEN, EDTA, HEDTA) was used with various concentrations of ZnSO_4_ (0.05–0.95 mm) in 50 mm HEPES, 100 mm NaCl, and 50 μm TCEP at pH 7.4 to maintain the free Zn^II^ concentration at a constant between subnano‐ and femtomolar levels. The concentration of zinc hook peptides in all studies was 5 μm. The sets of peptides in metal buffers (1.4 mL) were equilibrated over 48 h. Complex formation was measured by using FRET between Trp and Dns residues located at both ends of the zinc hook peptides. For that purpose, samples were excited at 290 nm, and spectra were collected in the range of 295–600 nm with a maximum emission of 543 nm. Anisotropy decay studies were performed with a DeltaFlex TCSPC Fluorimeter (Horiba) equipped with a Peltier‐thermostatted cell holder. All measurements were carried out at 25 °C in the buffer described above with 500 nm N‐terminally FAM‐labeled hook peptides. FAM‐labeled peptides (400 μL) were placed in a 1 mL all‐transparent quartz cuvette and excited with a linearly polarized laser from DeltaDiode DD‐485L (wavelength of (485±10) nm) through a 2 nm slit. Emission data were detected at 521 nm wavelength with a sequentially changing polarizer from 0 to 90° until accumulation of 10 000 peak difference at a 100 ns timescale was reached. Decay data were subsequently fitted with DAS6 software using two‐ or three‐exponential fitting, depending on the −log [M^II^]_free_ and metal‐to‐peptide equilibrium, as a VV+VH sum, VV−VH difference, and anisotropy. Given the very small targets (Hk14–Hk45 peptides), only difference spectra from reconvolution anisotropy analyses were taken into account because this approach most precisely accounts for instrument‐generated artifacts (Figures S14 and S15, Supporting Information). Zn^II^‐to‐Cd^II^ exchange experiments carried out for Hk45 with equimolar content of Cd_7_MT2a were performed accordingly, the only difference being faster equilibration times: 30 min for swap experiments and 36 h for competition experiments with metal chelators.

### Competitive titrations

The apparent formation constants (*K*_12_) of Cd^II^ biscomplexes with Hk14 and Hk45 peptides were determined fluorimetrically or spectropolarimetrically at 25 °C in the presence of Cd^II^ chelators HEDTA (log *K* ^7.4^=12.2), EDTA (log *K* ^7.4^=13.6), and TPEN (log *K* ^7.4^=15.2).^56^, ^60^ In the case of fluorescently labeled peptides, 5 μm Hk was incubated with 1 mm chelator with various total Cd^II^ concentrations in HEPES buffer with 50 μm TCEP. For CD monitoring, 5 μm Hk peptide was incubated with 25 μm chelator and 0–22.5 μm ZnSO_4_ in 20 mm Tris‐HCl buffer (pH 7.4, *I*=0.1 m from NaClO_4_) with 100 μm TCEP. Samples were incubated for 36 h under nitrogen. The free Cd^II^ concentration present in each sample after equilibration was calculated from the total chelator and metal concentrations, corrected for the Cd^II^ transferred to the Hk peptide during Cd(Hk)_2_ complex formation. Calculations were performed using the Hyperquad Simulation and Speciation Software (HySS2009).^51^ To obtain the apparent dissociation constants, we first determined the normalized isotherms corresponding to complex formation by fitting with Hill's equation.^33^ The obtained concentrations of free Cd^II^, referring to the half‐point complex saturation for which half of the total peptide is in the form of the Cd(Hk)_2_ complex and half is in the metal‐free form Hk, were subsequently used to calculate the apparent dissociation constants (*K*_12_).

### NMR spectroscopy

All spectra were measured with a DDR2 Agilent 600 MHz spectrometer equipped with a TRIAX probe. Assignment of ^1^H and ^13^C signals (Table S2, Supporting Information) was based on the previously described assignment of a peptide complexed with Zn^II^.^33^ 2D homonuclear TOCSY^88^ (mixing time 65 ms), NOESY^89^ (mixing time 150 ms), and heteronuclear ^1^H‐^13^C HSQC^90^ spectra recorded at 25 °C were used to confirm the assignment (relevant parameters of the NMR spectra are given in Table S3, Supporting Information). All chemical shifts in the ^1^H NMR spectra are reported with respect to external sodium trimethylsilylpropanesulfonate ([D_4_]DSS). Chemical shifts of the ^13^C spectra were referenced indirectly by using the 0.251449530 frequency ratios for ^13^C/^1^H.^91^ Processed spectra were analyzed with SPARKY software.^92^

### Isothermal titration calorimetry

The binding of Cd^II^ and Zn^II^ to Hk peptides was monitored using a nano‐ITC calorimeter (TA Waters, USA) at 25 °C with a cell volume of 1 mL. All experiments were performed in HEPES buffer (*I*=0.1 m from NaCl) at pH 7.4 with 0.25 mm TCEP used as a non‐metal‐binding reducing agent.^33^, ^36^ The Hk peptide (titrate) concentration was 0.1 mm, whereas the metal (titrant) concentration was 0.65 mm. After temperature equilibration, successive injections of the titrant were made into the reaction cell with 6.82 μL increments at 400 s intervals with stirring at 200 rpm, with the exception of the Zn^II^‐to‐Cd^II^ exchange experiment in which the titrant (CdSO_4_) concentration was 1 mm and the injection volume was 4.83 μL. Control experiments to determine the heats of titrant dilution were performed using identical injections in the absence of Zn^II^ and Cd^II^. The net reaction heat was obtained by subtracting the heat of dilution from the corresponding total heat of reaction. The titration data were analyzed using NanoAnalyze (version 3.3.0) and Origin (version 8.1) software.^75^

### Structure calculation

Determination of the structure of a symmetric dimer faces the well‐known problem of ambiguity of restraints. In principle each NOE cross‐peak can be a result of either contacts between protons of the same chain (intra) or different chains (inter) or both intra‐ and interchain contacts. Therefore, the structures of Hk14 peptide complexes with Cd^II^ and Zn^II^ ions were computed with Aria2 software,^93^ which can resolve ambiguous restraints in an iterative procedure. The parametrization of the coordination site with Zn^II^ ions was taken from the Aria2 program, with the exception of partial charges, which were computed using quantum chemistry methods applied to the model systems. For the complex with Cd^II^, bond lengths between sulfur and cadmium (set to 2.636 Å) were set to average distances obtained from the crystal structure, and partial charges were adjusted to match those obtained from quantum chemistry computations. For both peptides, partial charges were manually corrected so that the total charge of the coordination site (ion and four cysteine residues) is equal to −2|e|. The procedure of structure calculation consisted of nine simulated annealing cycles followed by refinement in an explicit solvent of 10 with the lowest energy out of 300 structures obtained from the last simulated annealing cycle. Each simulated annealing cycle consisted of 45 ps of high temperature dynamics (2000 K) followed by two cooling cycles: 90 ps of cooling to 1000 K and 72 ps of cooling to 50 K. Finally, 200 steps of structure optimization with the Powell algorithm were applied. The problem of ambiguity of applied restraints (inter‐ or intramonomer) was solved by the iterative procedure of NOE assignment implemented in the Aria2 software. The *C*2 symmetry of each complex was forced by application of special noncrystallographic symmetry restraints. Because of the ambiguity problem of NOE restraints, incorporation of additional hydrogen‐bond restraints was necessary. For each complex, two types of structure determination procedures were applied. Both of them utilized NOE restraints and differed in the number of applied hydrogen‐bond restraints. The first procedure (PROT) used hydrogen‐bond information obtained from experimentally determined protection factors,^33^ whereas in the second procedure (CRYST) it was assumed that the same hydrogen bonds are formed as in the crystal structure (PDB code: 1L8D). All applied hydrogen‐bond restraints are shown in Table S4. Notably, the CRYST method uses only one more hydrogen bond than the PROT method (backbone Gly3 O–Leu12 N). The average distance between two ends of the Hk14 peptide was estimated from 20 μs‐long molecular dynamics simulation. The peptide in extended conformation was built with the xleap program from the AMBER18 suite.^94^ Interactions between atoms were described with the ff14SB force field.^95^ The peptide was immersed in a truncated octahedron box filled with TIP3P model explicit water molecules. The system was minimized, heated, and equilibrated in an NPT ensemble followed by a 20 μs‐long NVT ensemble production run. The distance between ends was estimated as the average distance between α‐carbon atoms of the first and last amino acid residue. For error estimation, the trajectory was divided into four 5 μs‐long trajectories, and mean distances were calculated for each of them. The uncertainty was estimated as the standard deviation of mean values obtained from each window. Secondary structures were calculated for the whole trajectory with the DSSP method.^96^

## Conflict of interest

The authors declare no conflict of interest.

## Supporting information

As a service to our authors and readers, this journal provides supporting information supplied by the authors. Such materials are peer reviewed and may be re‐organized for online delivery, but are not copy‐edited or typeset. Technical support issues arising from supporting information (other than missing files) should be addressed to the authors.

Supplementary
