# Supplementary material for: Metal Exchange in the Interprotein ZnII‐Binding Site of the Rad50 Hook Domain: Structural Insights into CdII‐Induced DNA‐Repair Inhibition
Source: Chemistry. 2020 Jan 30;26(15):3297–313. doi: 10.1002/chem.201904942 (PMC7155053; doi:10.1002/chem.201904942)
Supplement: Supplementary file 1 — Supplementary [file CHEM-26-3297-s001.pdf]

CHEMISTRY
A European Journal

Supporting Information

Metal Exchange in the Interprotein Zn^{II}-Binding Site of the Rad50 Hook Domain: Structural Insights into Cd^{II}-Induced DNA-Repair Inhibition

Michał Padjasek,[a] Maciej Maciejczyk,[b] Michał Nowakowski,[c] Olga Kerber,[a] Maciej Pyrka,[b] Wiktor Koźmiński,[c] and Artur Krężel*[a]

chem_201904942_sm_miscellaneous_information.pdf

Materials

N,*N*-Diisopropylethylamine (DIEA), Fmoc-protected amino acids (Fmoc-Ala-OH·H_2O, Fmoc-Arg(Pbf)-OH, Fmoc-Asn(Trt)-OH, Fmoc-Asp(OtBu)-OH, Fmoc-Cys(Trt)-OH, Fmoc-Gln(Trt)-OH, Fmoc-Gln(Trt)-OH, Fmoc-Glu(OtBu)-OH, Fmoc-Gly-OH, Fmoc-His(Trt)-OH, Fmoc-Ile-OH, Fmoc-Leu-OH, Fmoc-Lys(Boc)-OH, Fmoc-Met-OH, Fmoc-Phe-OH, Fmoc-Pro-OH, Fmoc-Ser(tBu)-OH, Fmoc-Thr(tBu)-OH, Fmoc-Tyr(tBu)-OH, Fmoc-Val-OH, piperidine, *O*-(Benzotriazol-1-yl)-*N*,*N*,*N′*,*N′*-tetramethyluronium hexafluorophosphate (HBTU) and DL-dithiothreitol (DTT) were purchased from Iris Biotech GmbH. Trifluoroacetic acid (TFA), 1,2-ethanedithiol (EDT), thioanisole, anisole, triisopropylsilane (TIPS), COMU, guanidine hydrochloride (GdnHCl), 4-mercaptophenylacetic acid, tris(2-carboxyethyl)phosphine hydrochloride (TCEP), ethylenediaminetetraacetic acid (EDTA), HCl (trace metal grade), bis(β-aminoethyl ether)-*N*,*N*,*N'*,*N'*- tetraacetic acid (EGTA), *N*-carboxymethyl-*N′*-(2-hydroxyethyl)-*N*,*N′*-ethylenediglycine trisodium salt (Na_3-HEDTA), $NaClO_4{\cdot}H_2O$, $ZnSO_4{\cdot}7H_2O$ and $CdSO_4{\cdot}8/3H_2O$ were from Merck KGaA. Diethyl ether, acetic anhydride, dichloromethane (DCM), NaCl were purchased from Avantor Performance Materials Poland S.A. Chelex 100 resin from Bio-Rad, 4-(2-hydroxyethyl)piperazine-1-ethanesulfonic acid sodium salt (HEPES) from Bioshop, 5,5′-dithiobis-(2-nitrobenzoic acid) (DTNB) from TCI Europe N.V. TentaGel R RAM and TentaGel S-NH_2 resins from Rapp Polymere GmbH, dimethylformamide (DMF) and acetonitrile (MeCN) from VWR. All of the experiments were performed in chelexed buffers and solutions. All buffers were prepared with Milli-Q water obtained with a deionizing water system (Merck KGaA).

Table S1. Experimental and theoretical mass values of synthesized peptides and their sequences. *m* and *av* refer to monoisotopic and averaged masses, respectively. Ac-, -NH_2 , Dns and FAM stands for acetyl, amide, dansyl and fluorescein modification, respectively.

Peptide name	Peptide sequence	MW_{cal}	MW_{exp}
Hk4	Ac-CPVC-NH_2	461.2^{m}	461.4^{m}
Hk6	Ac-CPVCGR-NH_2	674.3^{m}	674.6^{m}
Hk8	Ac-KCPVCGRE-NH_2	931.4^{m}	931.7^{m}
Hk10	Ac-GKCPVCGREL-NH_2	1102.4^{m}	1103.3^{m}
Hk14	Ac-AKGKCPVCGRELTD-NH_2	1517.8^{av}	1518.4^{av}
FAM-Hk14	FAM-AKGKCPVCGRELTD-NH_2	1834.0^{av}	1835.4^{av}
Dns-Hk14-Trp	Dns-AKGKCPVCGRELTDW-NH_2	1893.9^{av}	1894.4^{av}
Hk27	Ac-EELKKAKGKCPVCGRELTDEHREELLS-NH_2	3140.0^{av}	3139.5^{av}
Hk31	Ac-AIEELKKAKGKCPVCGRELTDEHREELLSKY-NH_2	3615.4^{av}	3615.7^{av}
Hk37	Ac-LKTAIEELKKAKGKCPVCGRELTDEHREELLSKYHLD-NH_2	4322.9^{av}	4324.1^{av}
Hk45	Ac-KIGDLKTAIEELKKAKGKCPVCGRELTDEHREELLSKYHLDLNNS-NH_2	5164.8^{av}	5165.8^{av}
FAM-Hk45	FAM-KIGDLKTAIEELKKAKGKCPVCGRELTDEHREELLSKYHLDLNNS-NH_2	5481.1^{av}	5483.2^{av}
Hk130	RQLKEKLGDKSPEDIKKLLEELETKKTTIEEERNEITQRIGELKNKIGDLKTAIEELKK AKGKCPVCGRELTDEHREELLSKYHLDLNNSKNTLAKLIDRKSELERELRRIDMEIK RLTPLLTVAEQIRS	15217.8^{av}	$15\ 217.6^{av}$

Table S2. ^{1}H and ^{13}C chemical shifts (ppm) of Hk14 peptide complexed with Cd(II). Chemical shifts of second form when present are given in parentheses.

Residue/group	Atom name	^{1}H chemical shift	Atom name	^{13}C chemical shift
Acetyl group	H_M	1.996	C_M	24.42
1 Alanine	H_N<br>H_α<br>H_β	8.256<br>4.247<br>1.337	C_α<br>C_β	52.63<br>19.33
2 Lysine	H_N<br>H_α<br>H_β<br>H_γ<br>H_δ<br>H_ε	8.376<br>4.340<br>1.721; 1.845<br>1.482; 1.406<br>1.639<br>2.958	C_α<br>C_β<br>C_γ<br>C_δ<br>C_ε	56.1<br>33.34<br>35.29<br>28.97<br>42.18
3 Glycine	H_N<br>H_α	8.389<br>3.974	C_α	45.01
4 Lysine	H_N<br>H_α<br>H_β<br>H_γ<br>H_δ<br>H_ε	8.133<br>4.558<br>1.456; 1.395<br>1.284; 1.152<br>1.604<br>2.958	C_α<br>C_β<br>C_γ<br>C_δ<br>C_ε	54.6<br>24.87<br>24.92<br>29.06<br>42.18
5 Cysteine	H_N<br>H_α<br>H_β	8.758<br>4.581<br>2.764; 3.301	C_α<br>C_β	54.16<br>30.96
6 Proline	H_α<br>H_β<br>H_γ<br>H_δ	4.428<br>2.371; 2.029<br>2.127; 2.009<br>3.950; 4.316	C_α<br>C_β<br>C_γ<br>C_δ	64.31<br>32.47<br>27.14<br>51.77
7 Valine	H_N<br>H_α<br>H_β<br>$H_{\gamma1}$<br>$H_{\gamma2}$	8.876<br>4.06<br>2.005<br>0.964<br>0.931	C_α<br>C_β<br>$C_{\gamma1}$<br>$C_{\gamma2}$	65.26<br>31.87<br>21.87<br>23.12
8 Cysteine	H_N<br>H_α<br>H_β	7.952<br>4.981<br>2.886; 3.324	C_α<br>C_β	58.94<br>31.86
9 Glycine	H_N<br>H_α	7.873<br>3.837; 4.135	C_α	46.51
10 Arginine	H_N<br>H_α<br>H_β<br>H_γ<br>H_δ	8.048<br>4.376<br>1.783; 1.871<br>1.729<br>3.226; 3.094	C_α<br>C_β<br>C_γ<br>C_δ	56.43<br>31.56<br>27.89<br>43.66
11 Glutamic acid	H_N<br>H_α<br>H_β<br>H_γ	not assigned<br>4.234<br>2.264<br>2.298	C_α<br>C_β<br>C_γ	57.53<br>30.16<br>36.62
12 Leucine	H_N<br>H_α<br>H_β<br>H_γ	8.449<br>4.386 (4.387)<br>1.48; 1.557 (1.67, 1.61)	C_α<br>C_β<br>C_γ<br>$C_{\delta1}$	55.15 (55.46)<br>42.28 (42.24)<br>27.27 (27.07)<br>25.34 (25.03)

	$H_{\delta1}$<br>$H_{\delta2}$	1.475 (1.62)<br>0.795 (0.92)<br>0.859 (0.86)	$C_{\delta2}$	23.25 (23.46)
13 Threonine	H_N<br>H_α<br>H_β<br>$H_{\gamma2}$	8.231<br>4.344<br>4.242<br>1.176	C_α<br>C_β<br>$C_{\gamma2}$	61.70<br>69.91<br>21.51
14 Aspartic acid	H_N<br>H_α<br>H_β	8.322<br>4.581<br>2.683; 2.625	C_α<br>C_β	57.41<br>41.5
NH_2 group	H_1<br>H_2	7.474 (7.412)<br>7.048 (7.035)	-	-

Table S3. Relevant parameters of NMR spectra.

Spectrum	Number of scans	Complex points in indirect dimension	Spectral width (kHz)	Relaxation delay (s)
NOESY (150 ms mixing time)	96	512	12	1.5
TOCSY (65 ms mixing time)	8	256	12	1.5
^{1}H-^{13}C HSQC	64	512	12 ^{1}H/15 ^{13}C	1

Table S4. List of hydrogen bonds used in CRYST and PROT procedures of structure calculation. Hydrogen bond used only in CRYST calculations is indicated with boldface type. Hydrogen bonds removed in final calculations of second conformation of Cd(Hk)$_{14}$ with CRYST procedure are indicated with italic type.

Donor	Acceptor
Gly9 N	Cys5 O
Cys5 N	Arg10 O
**Leu12 N**	**Gly3 O**
Cys8 N	Cys5 S$_\gamma$
Val7 N	*Cys5 S$_\gamma$*
Arg10 N	*Cys8 S$_\gamma$*

Table S5. Average energies and number of violated NOE and H-bond restraints in all calculated families of structures. In parentheses are mean energies of eight low-energy structures obtained after removal of two structures with severely violated NOE restraints.

	violations	$Zn(Hk14)_2$	$Cd(Hk14)_2$ conformation 1	$Cd(Hk14)_2$ conformation 2	$Cd(Hk14)_2$ conformation 2 w/o weak N-S H-bonds
CRYST	NOE (kcal/mol)	6.5	7.7	32.7	13.1
	H-bond (kcal/mol)	2.0	7.7	166.2	4.4
	total (kcal/mol)	8.5	15.4	198.9	17.5
	Average number of NOE violated between 0.1 - 0.3 Å	5.7	4.6	9.8 and 1.6 violations > 0.3 Å	6.4 and 0.4 violations > 0.3 Å
	Average number of H-bonds violated between 0.1 - 0.3 Å	1.3	4.5	6.5 and 3.8 violations > 0.3 Å	2.9
PROT	NOE (kcal/mol)	9.5	5.0	73.2 (32.3)	-
	H-bond (kcal/mol)	5.1	2.2	3.1 (3.0)	-
	total (kcal/mol)	14.6	7.2	76.3 (35.3)	-
	Average number of NOE violated between 0.1 - 0.3 Å	3.9	2.4	13.2 and 1.9 violations > 0.3 Å	-
	Average number of H-bonds violated between 0.1 - 0.3 Å	4.2	1.1	2.3	-

Table S6.. Thermodynamic parameters of hook peptides titrated with Zn(II) or Cd(II) derived from spectroscopic approaches conducted during the scope of these studies.

Hook peptide	$\Delta G°_{Zn(II)}$ (kcal/mol)	$\Delta G°_{Cd(II)}$ (kcal/mol)
Hk4	-20.37	-23.40
Hk6	-22.43	-25.91
Hk8	-24.26	-27.34
Hk10	-25.43	-28.21
Hk14	-26.18	-28.87
Hk27	-26.97	-29.96
Hk31	-27.90	-30.83
Hk37	-28.22	-30.88
Hk45	-28.29	-30.88
Hk130	-28.40	-31.01

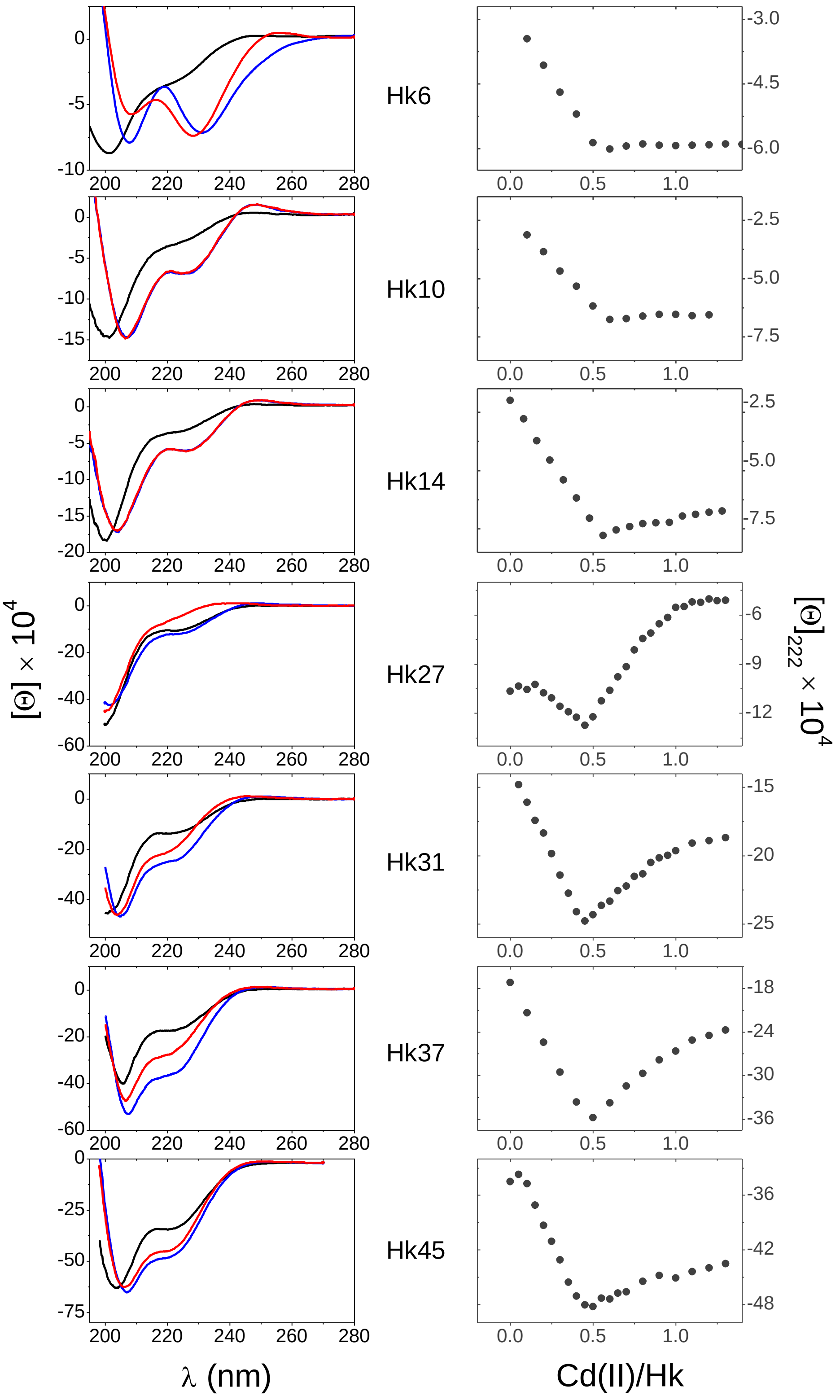

Figure S1. CD spectra of Hk6-45 titrated with Cd(II). Panel on the left hand side shows molar ellipticity changes (in deg·cm^2·dmol^{-1}) in increasing wavelength, where black line represents 0 Cd(II) molar equivalents over hook peptide, blue line 0.5 Cd(II) and red line 1 Cd(II) molar equivalent. Right-hand side panel illustrates molar ellipticity changes at 240 nm as a function of Cd(II)/Hk molar ratio.

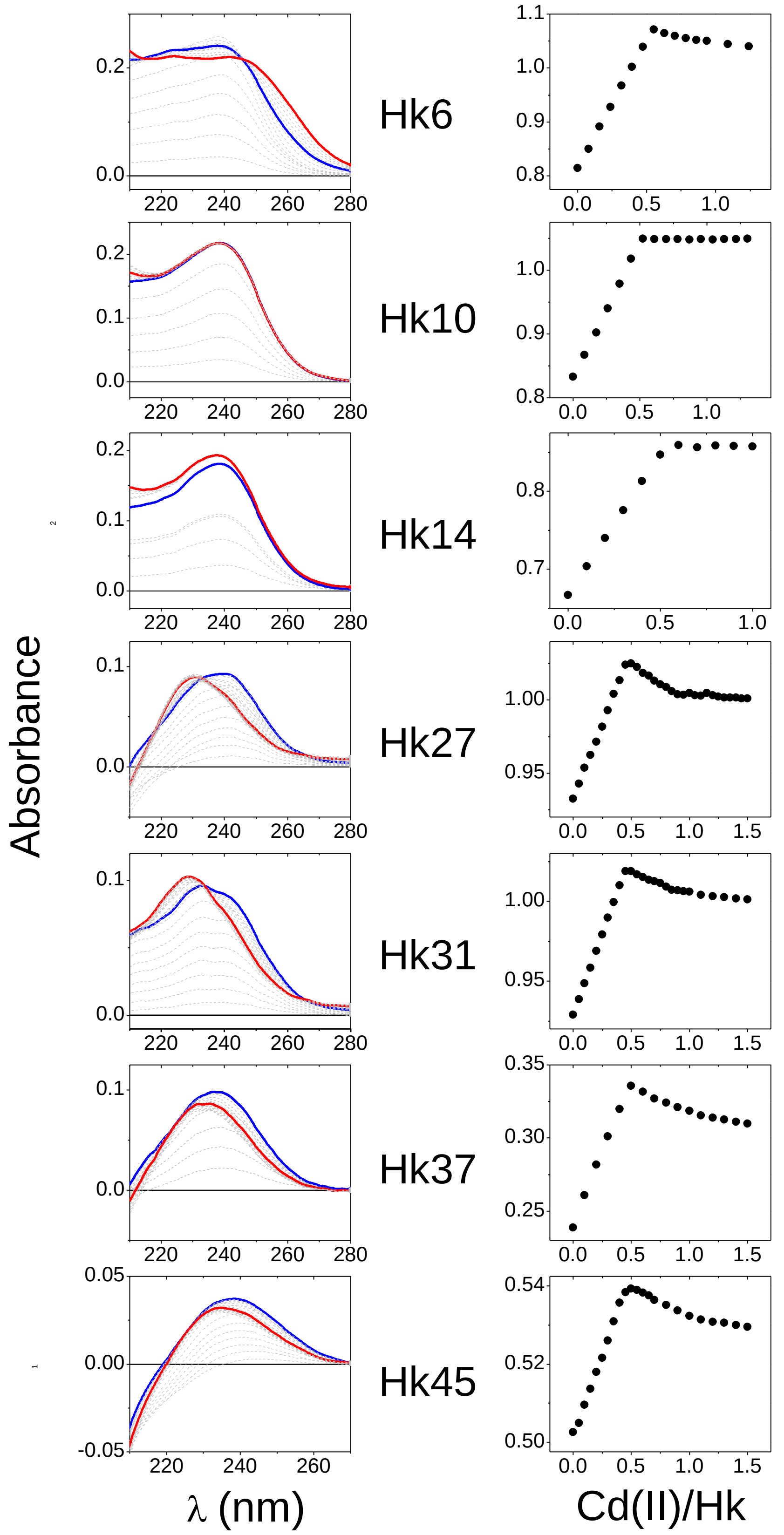

Figure S2. Differential UV-absorption spectra of Cd(II)-titrated Hk6-45 (left-hand side) and absorption in 240 nm represented as a function of Cd(II)/Hk molar ratio (right-hand side). Black line illustrates apo form, blue line 0.5 and red line 1 molar equivalent of Cd(II) over hook peptide.

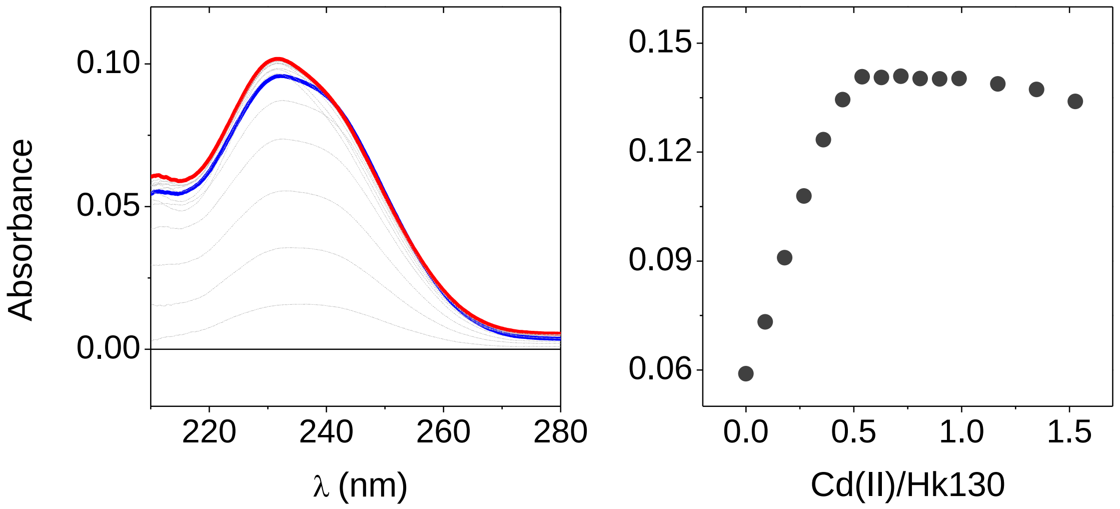

Figure S3. Differential UV-absorption spectra of Cd(II)-titrated Hk130 (left-hand side) and absorption in 240 nm represented as a function of Cd(II)/Hk130 molar ratio (right-hand side). Black line illustrates apo form, blue line 0.5 and red line 1 molar equivalent of Cd(II) over hook peptide.

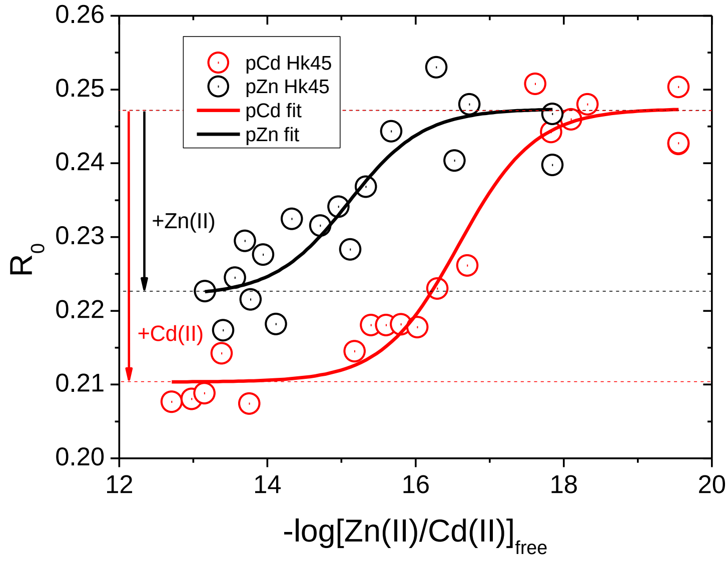

Figure S4. Anisotropy decay-derived initial anisotropy (R_0) changes represented as a function of free Zn(II)/Cd(II) concentration. Black points and line illustrate Hk45 initial anisotropy changes in $-\log[Zn(II)]_{free}$ range and red ones $-\log[Cd(II)]_{free}$, where points stand for experimental values and lines show Hill equation fits. Arrows indicate overall parameter change from apo- to holo- hook domain for Cd(II) and Zn(II) complexation in red and black, respectively.

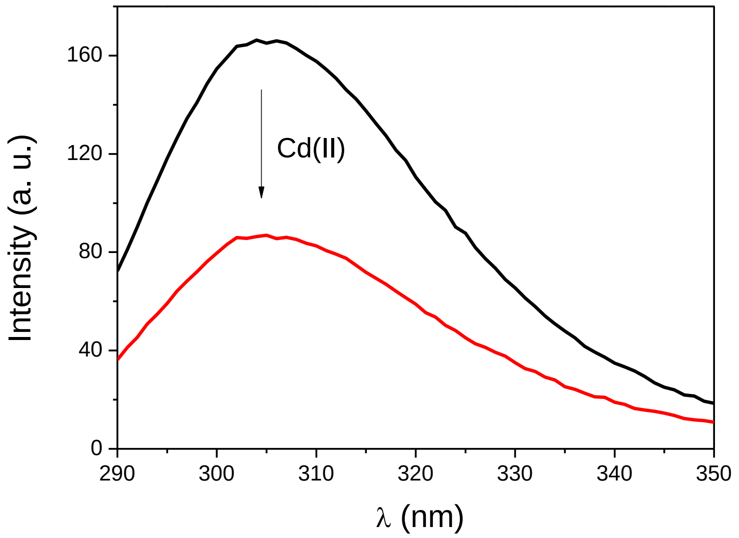

Figure S5. Tyrosine emission (λ_{ex} = 275 nm) changes of 4 μM Hk130 fragment of metal free (black line) and cadmium ML_2 complex (red line) in 20 mM Tris-HCl, pH 7.4, I = 0.1 M.

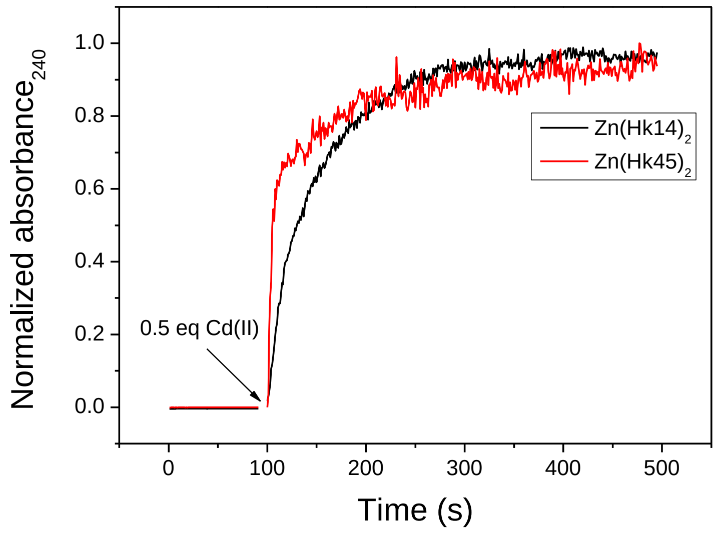

Figure S6. Cd(II)-binding kinetics of 10 μM $Zn(Hk14)_2$ (black line) and $Zn(Hk45)_2$ (red line) monitored as the absorption increase at 240 nm wavelength in time. Increasing absorption corresponds to formation of Cd-S bond and LMCT bands with absorption maximum around 240 nm. The Cd(II)-binding of $Zn(Hk14)_2$ is a first-order reaction with rate constant calculated to be ~0.015 s^{-1}. In case of $Zn(Hk45)_2$ the reaction is much faster with reaction order and rate constant impossible to be determined accurately under used experimental settings.

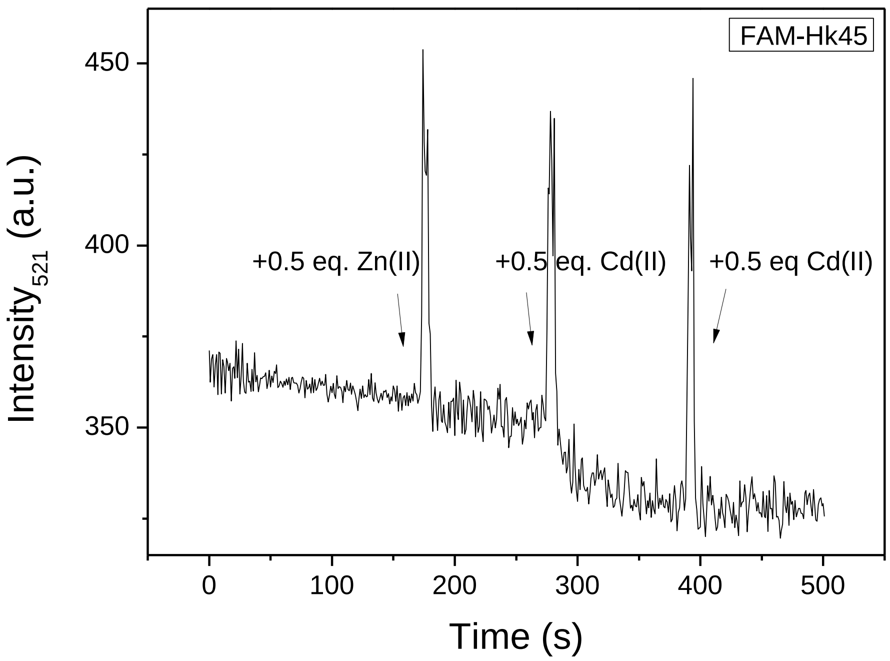

Figure S7. Zn(II)-to-Cd(II) exchange kinetics of 5 μM FAM-labeled Hk45 monitored as a FAM emission change (521 nm) in time. Arrows indicate titration steps of 0.5 equivalents of Zn(II) and Cd(II). Cd(II) titration resulted in emission decrease, while subsequent addition showed no emission change indicating that fluorescence change is not artificial.

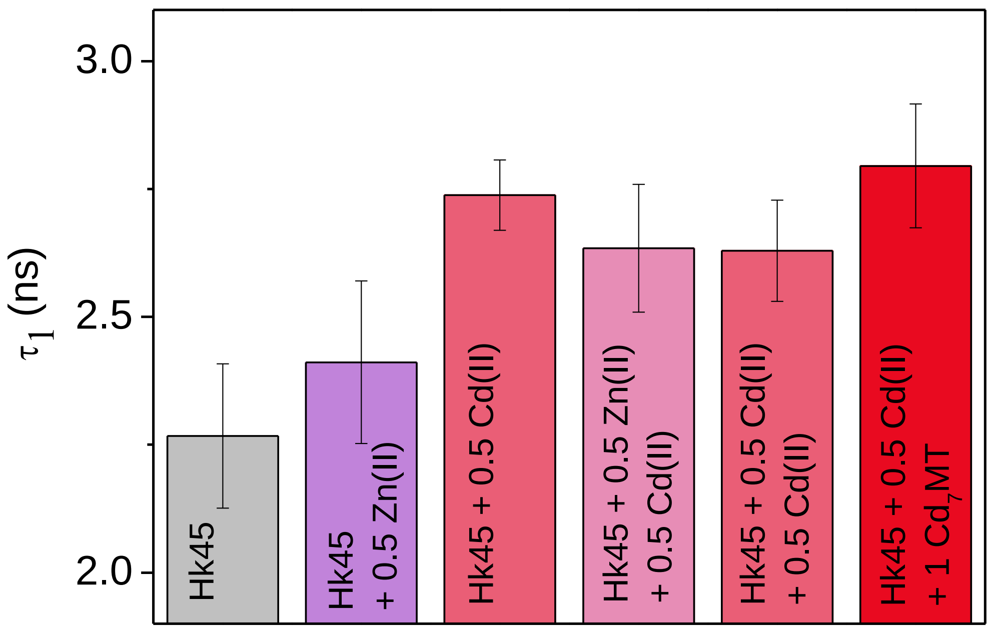

Figure S8. Rotational correlation time values of directly titrated Hk45 with Zn(II), Cd(II) and Cd_7MT2 with standard deviation errors from 2-exponential fitting of reconvolution-analyzed data. Bars from left to right represent: apo form (grey), hook with 0.5 molar equivalent of Zn(II) (purple), 0.5 equivalent of Cd(II) (pale red), 0.5 equivalent of Zn(II) and subsequent 0.5 equivalent of Cd(II) (pale purple), 0.5 equivalent of Cd(II) and subsequent portion of Cd(II) (pale red), 0.5 equivalent of Cd(II) and subsequent 1 equivalent of Cd_7MT2 (red).

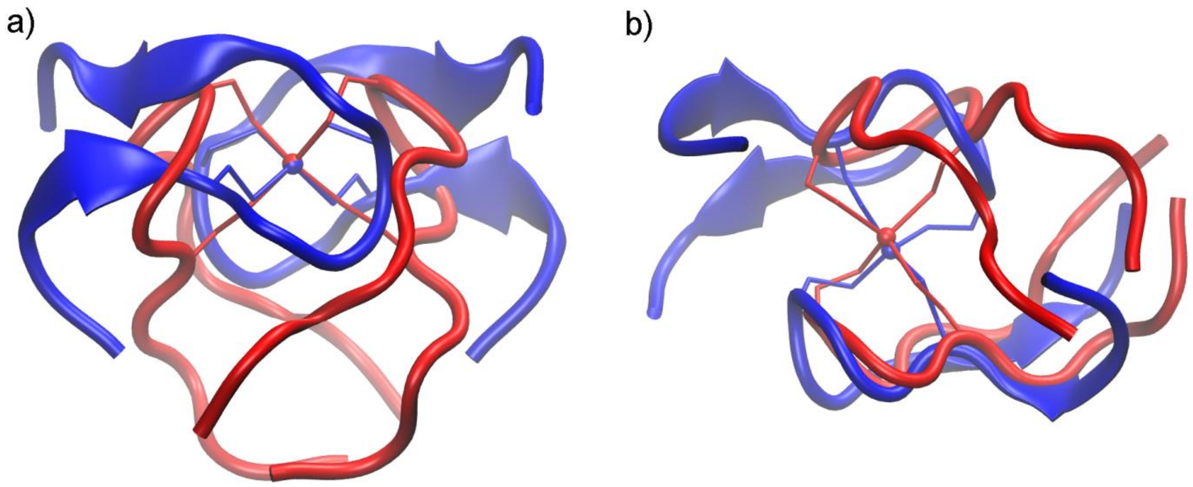

Figure S9. Structural alignment of lowest energy structures of $Zn(Hk14)_2$ (blue) and $Cd(Hk14)_2$ (red). A) Structures aligned *via* Zn(II)/Cd(II)-coordinating sulfur atoms. B) Structures aligned *via* single peptide chain.

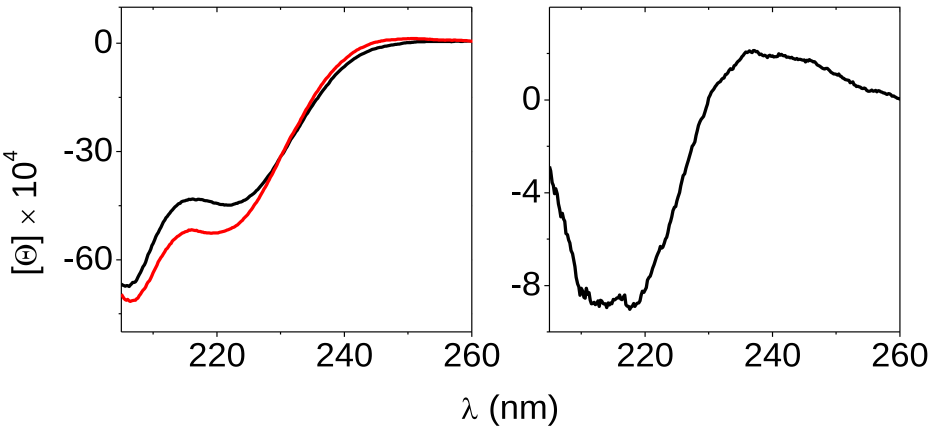

Figure S10. Left-hand side: CD spectra of $Zn(Hk45)_2$ (black line) and $Cd(Hk45)_2$ red line represented as molar ellipticity changes (in deg·cm^2·dmol^{-1}) in UV range. Right-hand side: $Cd(Hk45)_2$ – $Zn(Hk45)_2$ subtracted spectrum showing regions of major differences in secondary structure.

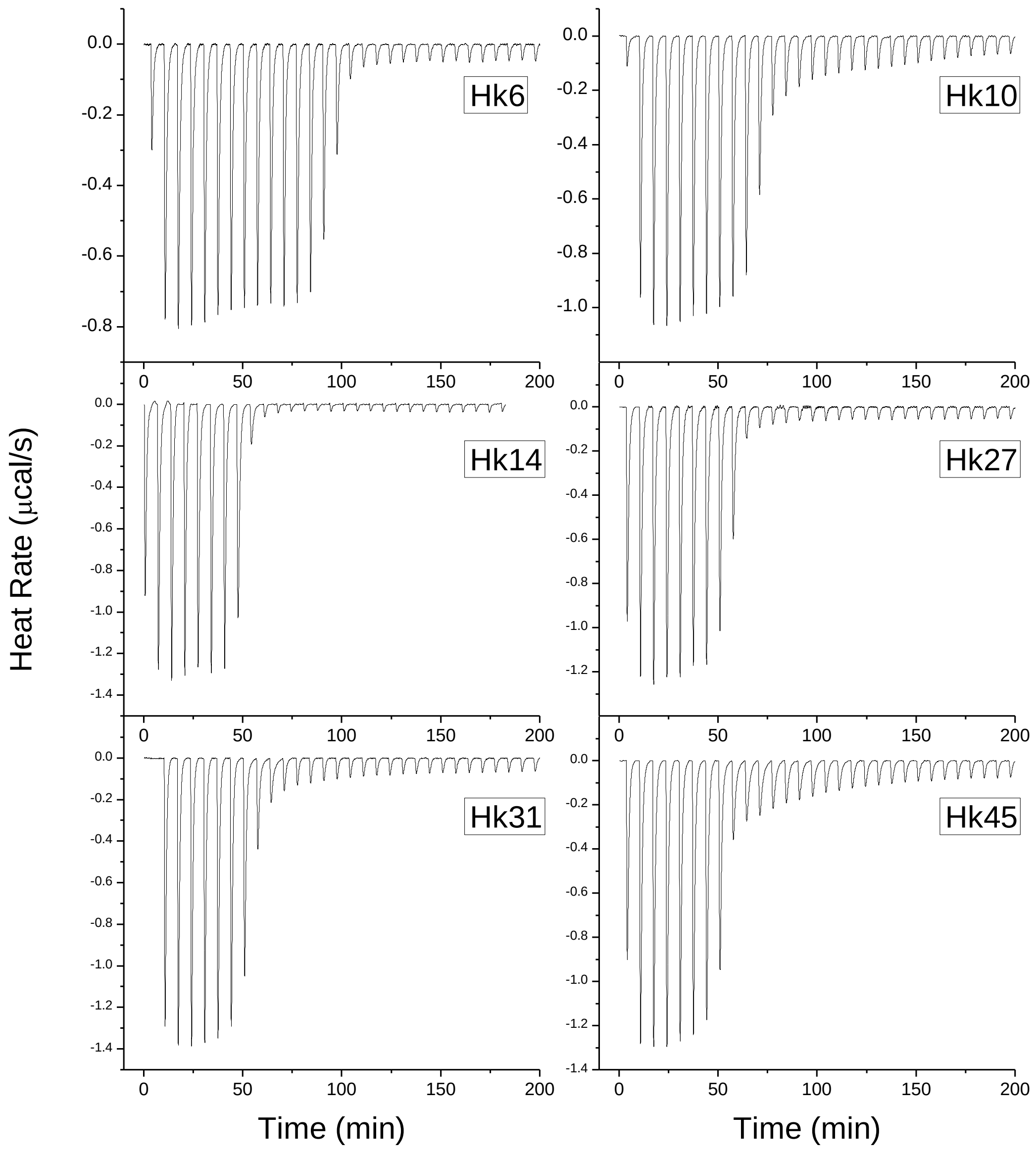

Figure S11. ITC titration of Hk6-45 peptides with Cd(II). Corrected heat rates by baseline. subtraction are represented as a function of time. Heat values were taken to determine ΔH_{ITC} used for subsequent ΔS^{o} calculations, while affinity and ΔG^{o} were calculated based on potentiometric and competition analyses.

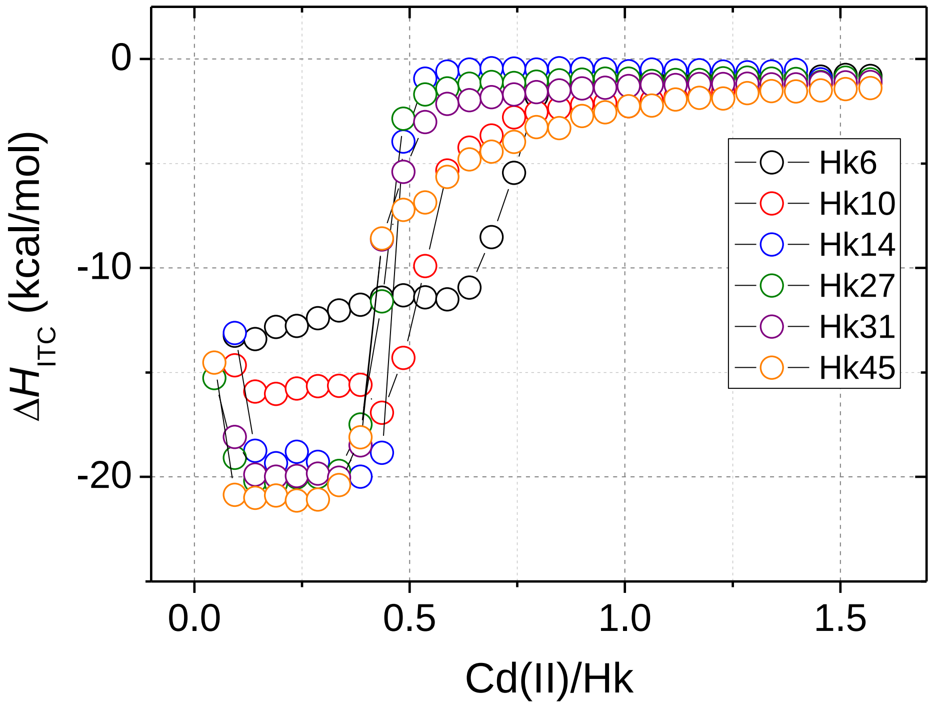

Figure S12. Enthalpy heat values from ITC experiment of Hk6-45 titrated with Cd(II) presented as a function of Cd(II)/hook molar ratio. Data were not fitted and only absolute heat values were taken into further calculations, with affinity constants derived from potentiometric and competition analyses.

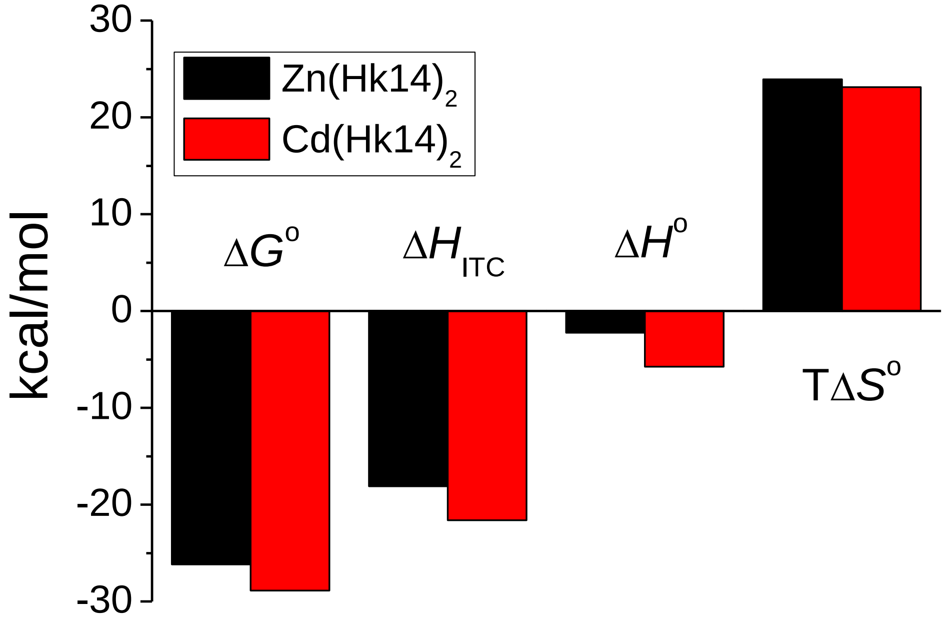

Figure S13. Thermodynamic parameters of Zn(II)- and Cd(II)-titrated Hk14 derived during the course of these studies. Black and red bars represent Zn(II) and Cd(II) titrations, respectively.

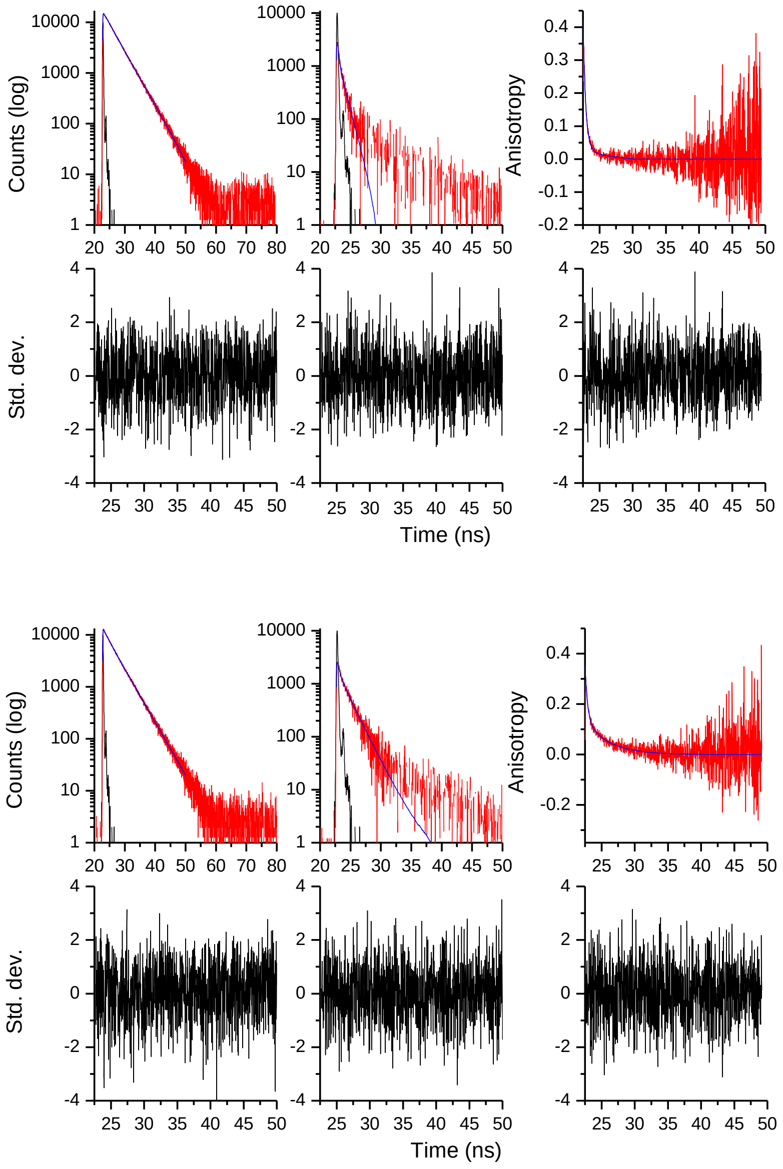

Figure S14. Exemplary anisotropy decay analysis results of a) Cd(FAM-Hk14)$_2$ and b) Cd(FAM-Hk45)$_2$ represented as log of photon counts (or anisotropy) per time in ns. Upper panel from left to right: sum of VV and VH decay data, difference of VV and VH data and anisotropy data. Black line represents IRF data points, red line represents sample's experimental data and blue line represents 2-exponential fit. Lower panel represents standard deviation of 2-exponential fits performed on appropriate data from upper panel graphs.

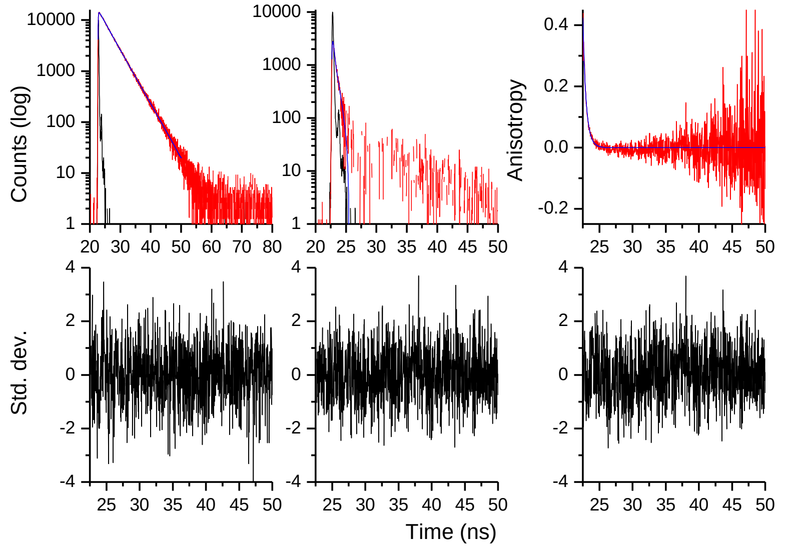

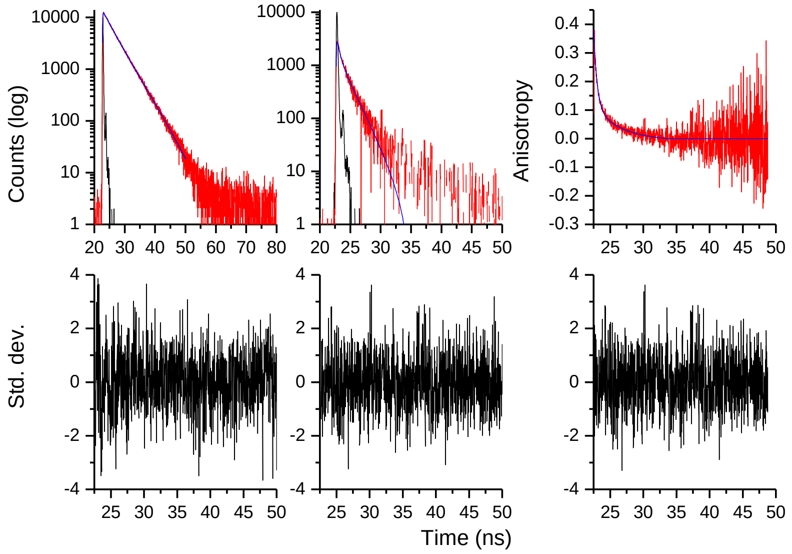

Figure S15. Exemplary anisotropy decay analysis results of a) Zn(FAM-Hk14)$_2$ and b) Zn(FAM-Hk45)$_2$ represented as log of photon counts (or anisotropy) per time in ns. Upper panel from left to right: sum of VV and VH decay data, difference of VV and VH data and anisotropy data. Black line represents IRF data points, red line represents sample's experimental data and blue line represents 2-exponential fit. Lower panel represents standard deviation of 2-exponential fits performed on appropriate data from upper panel graphs.
